# Local coordination geometry within cobalt spinel oxides mediates photoinduced polaron formation†

**DOI:** 10.1039/d5sc01909e

**Published:** 2025-05-12

**Authors:** Erica P. Craddock, Jacob L. Shelton, Michael T. Ruggiero, Kathryn E. Knowles

**Affiliations:** a Department of Chemistry, University of Rochester Rochester NY 14627 USA kknowles@ur.rochester.edu

## Abstract

Understanding the photophysics of transition metal oxides is crucial for these materials to realize their considerable potential in applications such as photocatalysis and optoelectronics. Recent studies suggest that formation of localized excited states consisting of polarons (quasi-particles comprising a charge carrier strongly coupled to a proximal lattice distortion) plays a crucial role in the photophysics of these materials. Cobalt-containing spinel oxides (Co_3_O_4_ and ZnCo_2_O_4_) offer a unique opportunity to investigate the influence of local geometry, and cation inversion on photoinduced polaron formation. Here, we use Hubbard-corrected density functional theory (DFT + *U*) paired with resonance Raman and temperature-dependent optical spectroscopies to demonstrate that low-energy transitions observed in Co_3_O_4_ are associated with d–d transitions involving cobalt ions occupying tetrahedral sites within the spinel lattice. These low-energy optical transitions exhibit strong coupling to phonon modes associated with tetrahedral sites. Replacing most tetrahedral cobalt ions with zinc produces the slightly inverted ternary spinel material, ZnCo_2_O_4_, in which we observe a phonon-coupled optical transition that occurs at the same energy as observed in Co_3_O_4_. We propose that these phonon-coupled optical transitions enable direct access to a polaronic state upon photoexcitation; however, the intensity of this optical transition depends on temperature in Co_3_O_4_, whereas no significant temperature dependence is observed in ZnCo_2_O_4_. We therefore hypothesize that in Co_3_O_4_ the mechanism of polaron formation is coupling of the optical transition to dynamic, thermally-gated lattice distortions, whereas, in ZnCo_2_O_4_, the transition couples to static lattice defects that arise from the presence of a small population of tetrahedrally-coordinated cobalt ions.

## Abstract 2

Comparison of the optical properties of Co_3_O_4_ and ZnCo_2_O_4_ elucidates fundamental differences in mechanisms of photoinduced polaron formation based on the presence or absence of substitutional lattice defects arising from cation inversion.

## Graphical abstract

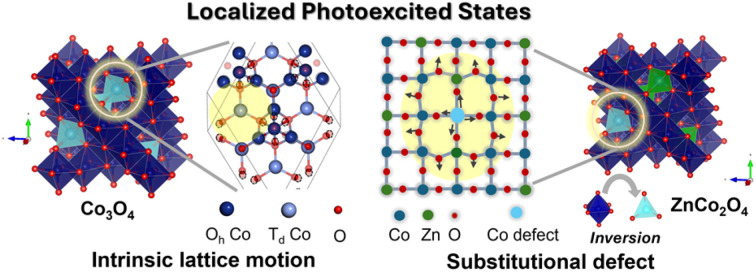

## Introduction

Transition metal oxides have promising characteristics for solar energy conversion technologies because of their visible band gap energies, stability, abundance, cost-effectiveness and low toxicity.^1,2^ However, these materials contain weakly dispersive bands originating from the metal 3d orbitals that lead to low charge-carrier mobility,^3,4^ rapid charge recombination,^5,6^ and formation of localized, “self-trapped” states near the band-edges.^7^ These states, which comprise one or more localized charge carriers coupled to proximal lattice distortions arising from one or more phonons, are known as polarons.^7,8^ Small polarons, first described by Holstein,^9,10^ are described by a short-range carrier–phonon interaction (radius_polaron_ ∼ lattice parameter) and carrier mobility that increases with increased temperature.^11^ Conversely, large polarons have long-range carrier–phonon interactions (radius_polaron_ ≫ lattice parameter) with carrier mobility that decreases with increased temperature.^12,13^ Many transition metal oxides are reported to host small polarons,^7,14–19^ and in many cases the performance of these materials for electrocatalytic, photoelectrocatalytic, and optoelectronic applications is impacted by small polaron formation.^20–25^

**Fig. 1:**
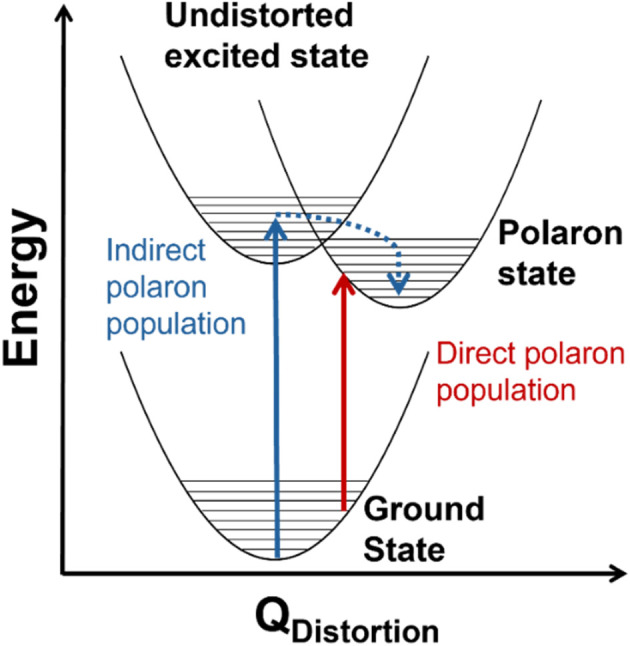
Conceptual configuration coordinate diagram depicting mechanisms of photoexcited polaron formation. The red arrow illustrates direct photoexcitation from a thermally distorted ground state into a polaronic state and the blue arrow shows relaxation into a polaronic state *via* an undistorted ground state.

Although conductivity studies have contributed to a thorough understanding of polaron transport mechanisms in transition metal oxides,^11,15,17,26,27^ there is still a limited understanding of the mechanisms by which polarons form in photoexcited states. In hematite (α-Fe_2_O_3_), for example, one proposed mechanism involves indirect population of polaron states *via* relaxation from an initially excited, higher-energy non-polaronic state.^20,28^ Our group recently identified an additional mechanism involving the direct population of polaronic excited states at room temperature upon band-edge excitation in α-Fe_2_O_3_.^29,30^Fig. 1 depicts the difference between indirect and direct population of polaronic states *via* photoexcitation. Importantly, optical population of polaronic states in α-Fe_2_O_3_ is proposed to occur even in a pristine, defect-free lattice: the optical transitions couple to intrinsic, thermally-activated phonon distortions within the crystal.^29,30^ There is also evidence of polaron formation arising from charge carriers coupling to intrinsic lattice distortions in rutile TiO_2_ and LiNbO_4_.^16,31^ These self-trapping mechanisms differ from other descriptions of small polarons forming *via* charge carriers coupling to dopants or lattice defects.^32–35^ Distinguishing the mechanism of photoinduced polaron formation (mediated by dynamic thermally activated lattice distortions or static lattice defects) is fundamentally important to the development of strategies for engineering metal oxide materials for photoapplications.

**Fig. 2:**
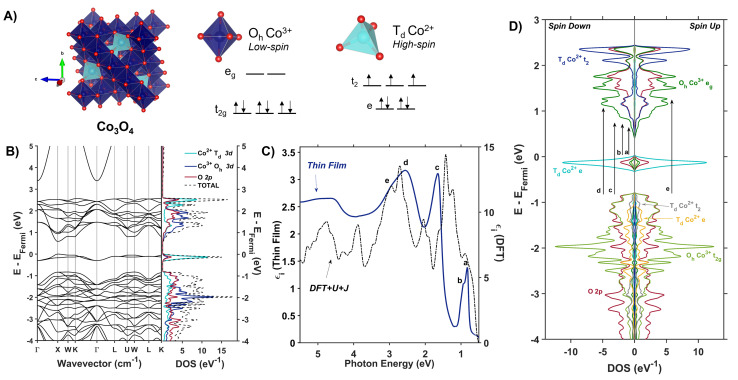
(A) The Co_3_O_4_ unit cell (*Fd*3̄*m*) with associated d-splitting diagrams for Co^3+^ (O_h_) and Co^2+^ (T_d_). (B) Electronic band structure and projected density of states of Co_3_O_4_ calculated with Hubbard-corrected DFT. (C) Plot of experimentally (solid blue line) and computationally (dashed black line) determined imaginary dielectric spectra of Co_3_O_4_. Labels a–e correlate with arrows in (D), the spin-symmetrized projected density of states arising from the primitive cell of Co_3_O_4_.

Spinel oxides (AB_2_O_4_) offer a unique opportunity to understand how the mechanism of photoinduced polaron formation depends on orbital composition of the band edge, coordination geometry of metal centers, and the presence of substitutional defects. These materials are mixed-valent with *Fd*3̄*m* symmetry and two different site symmetries for metals: tetrahedral (T_d_) and octahedral (O_h_). Ternary spinel oxides, in which A and B are different metals, are described by an inversion parameter *x* (0.0 < *x* < 1.0) that quantifies the percentage of the A cations that occupy octahedral sites. When *x* = 0.0, meaning all of the A cations are in tetrahedral sites, the spinel is considered “normal,” whereas when *x* = 1.0, meaning all of the A cations are in octahedral sites, the spinel is fully inverted. Intermediate values of *x* correspond to population of A cations in both tetrahedral and octahedral sites. Spinel oxides containing cobalt are of particular interest because of the difference in crystal field splittings of Co^2+^ T_d_ and Co^3+^ O_h_ (Fig. 2A). Transitions between the e and t_2_ states within the T_d_ sites are allowed by the Laporte selection rule whereas this rule forbids transitions between the t_2g_ and e_g_ states within the O_h_ sites. Normal Co_3_O_4_, which contains both Co^2+^ T_d_ and Co^3+^ O_h,_ exhibits optical transitions associated with localized d–d transitions at 0.8, 0.9 and 1.6 eV, in addition to an optical transition at 2.5 eV associated with a ligand-to-metal-charge transfer (LMCT) transition.^36–40^ Normal ZnCo_2_O_4_, with only Co^3+^ O_h_, is reported to have only the LMCT-type transition at 2.5 eV.^27^ The coordination geometry of cobalt in spinel oxides thus impacts their optical spectra; we aim to understand how this coordination geometry impacts photoinduced polaron formation.

The formation of small polarons in Co spinel oxides (Co_3_O_4_ and ZnCo_2_O_4_) has been inferred from the observation of thermally activated charge transport in these materials;^26,27^ however, as with other transition metal oxides, descriptions of photoinduced polarons in these materials are scarce. Transient absorption (TA) studies of Co_3_O_4_ have reported that the strongest TA signal is induced by thermal effects,^41,42^ which mirrors the behavior of hematite, a material known to undergo photoinduced polaron formation.^29,30,43,44^ In other work using extreme ultraviolet (XUV) spectroscopy, Zhang, *et al.* describe indirect formation of small polarons from self-trapped photocarriers in Co_3_O_4_.^45^ Using a combined approach of resonance Raman spectroscopy, temperature-dependent optical spectroscopy and Hubbard-corrected density functional theory, we investigate the influence of cobalt coordination geometry on photoinduced polaron formation in Co_3_O_4_ and ZnCo_2_O_4_. We report evidence that T_d_ Co in Co_3_O_4_ mediates resonance Raman enhancement of specific phonon modes, indicating the presence of phonon-coupled optical transitions that lead to polaron formation. Parallel studies of partially inverted ZnCo_2_O_4_, in which the majority of the T_d_ Co ions are replaced with Zn, confirm the involvement of T_d_ Co in phonon-coupled transitions; however, in ZnCo_2_O_4_ these transitions do not depend on temperature. This observation suggests that photoinduced polarons in ZnCo_2_O_4_ form at static defects rather than dynamic lattice distortions. The fundamental understanding of photophysical properties as a function of coordination geometry presented here is crucial to designing transition metal oxides for optical applications.

## Results and discussion

### Assignment of the optical spectra of Co_3_O_4_

Co_3_O_4_ adopts a spinel crystal structure (space group *Fd*3̄*m*) with two different metal sites: Co^3+^ ions occupy octahedral sites and Co^2+^ ions occupy tetrahedral sites (Fig. 2A). The two coordination sites give rise to two distinct crystal field splittings of the 3d orbitals as shown in Fig. 2A. Transitions between the e and t_2_ states within the T_d_ sites are allowed by the Laporte selection rule whereas this rule forbids transitions between the t_2g_ and e_g_ states within the O_h_ sites. The unpaired electrons of adjacent T_d_ Co^2+^ atoms in Co_3_O_4_ are antiferromagnetically coupled.^46^ Density Functional Theory with Hubbard and Hund corrections (DFT + *U* + *J*) was used to calculate the ground-state electronic structure of Co_3_O_4_ within the Born–Oppenheimer approximation, in which nuclear motion is neglected. Hubbard (electron correlation correction)^47^ and Hund (local magnetization correction)^48^ corrections are used in highly correlated materials such as Co_3_O_4_ to mitigate self-interaction errors.^30^ Using a linear response method,^30,47^*U* and *J* parameters were calculated for Co_3_O_4_ from first principles *via* perturbation of the local environments of open-shell ions (T_d_ Co^2+^ and O_h_ Co^3+^ in the case of Co_3_O_4_, see ESI† for more computational details). The DFT + *U* + *J*-computed band structure and projected density of states (pDOS) shown in Fig. 2B indicate that 3d orbitals associated with Co^2+^ T_d_ atoms are the primary contributors to an energetically isolated band at the valence band maximum (VBM), whereas both Co^2+^ T_d_ and Co^3+^ O_h_ 3d orbitals contribute to the conduction band minimum (CBM). These computed results suggest isolated valence bands with Co^2+^ T_d_ character participate in the low-energy transitions observed in the experimental imaginary dielectric spectrum at 0.82 and 0.92 eV (labeled a, b in Fig. 2C).

Fig. 2C plots the DFT + *U* + *J*-computed dielectric spectrum overlaid with the experimental dielectric spectrum extracted from transmission and reflection spectra measured from a 53.7-nm thick Co_3_O_4_ film (see ESI† for details of dielectric spectrum determination and powder X-ray diffraction pattern). We applied a rigid shift of +0.3 eV to all conduction band eigenvalues to bring the computed dielectric function into alignment with the measured spectrum. Herein, this shift is applied to all electronic band diagrams and electronic density of states plots of Co_3_O_4_. In order to fulfill the *f*-sum rule governing total oscillator strength, all computed optical spectra are subsequently renormalized by a factor of (1 − (0.3 eV/*ħω*)).^49,50^ The computed single-particle dielectric spectrum shows good agreement with the measured spectrum, confirming the features at 0.82, 0.92 and 1.64 eV involve Co^2+^ T_d_ bands (a–c). From the spin-resolved density of states separated into band contributions from t_2g_ and e_g_ (O_h_), e and t_2_ (T_d_) and O 2p (Fig. 2D), optical transitions are assigned while also considering spatial wavefunction overlap. The onsets of the conduction bands derived from O_h_ Co^3+^ e_g_, T_d_ Co^2+^ t_2_, and O 2p orbitals occur at the same energy; however, the largest projected density of states comes from O_h_ Co^3+^ e_g_. Thus, when considering transitions from T_d_ Co^2+^ e to the conduction band, these DFT + *U* + *J* computations exhibit no energetic difference among transitions to O_h_ Co^3+^ e_g_, T_d_ Co^2+^ t_2_, and O 2p. The oscillator strength of the experimental dielectric peak at 0.82 eV is higher than the 0.92 shoulder, indicating the higher likelihood of the 0.82-eV transition; however, the shoulder is not resolved in the imaginary dielectric computed by DFT + *U* + *J*. Therefore, to assign this shoulder, we qualitatively assessed the spatial overlap of orbital wavefunctions by mapping the contributions of specific atoms to specific projected densities of states (Fig. S10†). This approach reveals that the most probable transition contributing to the 0.82 eV feature is an intra-atomic transition of Co^2+^ T_d_ that satisfies the spin transition selection rule (Fig. S10A and B†). The spatial overlap of the orbitals involved in an intra-atomic transition is greater than the overlap of orbitals between a T_d_ Co and an O_h_ Co (inter-sublattice charge transfer), further supporting the assignment of the 0.82-eV optical transition to an intra-atomic d-to-d transition in T_d_ Co^2+^ (e → t_2_). From the spin-resolved density of states combined with our spatial overlap analysis, the feature at 0.92 eV is best described as a charge transfer transition from the tetrahedral sublattice to the octahedral sublattice (T_d_ Co^2+^ e → O_h_ Co^3+^ e_g_). Similarly, the transition at 1.62 eV can be described as an inter-sublattice charge transfer from O_h_ Co^3+^ t_2g_ → T_d_ Co^2+^ t_2_. The covalency between T_d_ Co^2+^ and O 2p, as well as O_h_ Co^3+^ and O 2p, allows these metal-to-metal (inter-sublattice charge transfer) transitions to occur. The feature at 2.55 eV and its shoulder at 2.90 eV arise from ligand-to-metal charge transfer (LMCT) type transitions. The assignment of these two LMCT transfers is resolved from comparing the experimental spectrum of Co_3_O_4_ with ZnCo_2_O_4_, which has majority O_h_ Co^3+^ (Fig. S12†). It becomes apparent that the O 2p → O_h_ Co^3+^ e_g_ transition is slightly higher in energy than the O 2p → T_d_ Co^2+^ t_2_ transition, leading to the following assignments: O 2p → T_d_ Co^2+^ t_2_ (2.55 eV) and O 2p → O_h_ Co^3+^ e_g_ (2.90 eV). All transitions are spin-conserved (Fig. 2D and Table 1).

**Table 1:** Assignment of optical transitions in Co_3_O_4_

Peak center (eV)	Label	Transition	Description
0.82	a	T_d_ Co^2+^ e → t_2_	Intra-atomic transition
0.92	b	T_d_ Co^2+^ e → O_h_ Co^3+^ e_g_	Inter-sublattice charge transition
1.64	c	O_h_ Co^3+^ t_2g_ → T_d_ Co^2+^ t_2_	Inter-sublattice charge transition
2.55	d	O 2p → T_d_ Co^2+^ t_2_	Ligand-to-metal charge transition
2.90	e	O 2p → O_h_ Co^3+^ e_g_	Ligand-to-metal charge transition

The electronic density of states and band structure of Co_3_O_4_ have been previously calculated using many approaches including, but not limited to GGA + *U*, hybrid functional PBE0, range-separated exchange–correlation functional HSE06, and many-body Green's function GW approximation.^27,51,52^ Commonly, the density of states shows some degree of Co 3d and O 2p wavefunction overlap at the band edges; however, depending on the computational approach, the band gap varies from 0.78 to 1.6 eV.^27,51,52^ Singh, *et al.* explored many different DFT approaches to calculating the electronic structure of Co_3_O_4_, including PBE, PBE + *U*_eff_, HSE06, and many-body Green's function using the GW approximation (Sc-GW0).^52^ Of these, the Sc-GW0 method produces the most accurate representation of the electronic structure of Co_3_O_4_ based on computed electronic bands, density of states, and absorption spectra.^52^ Although the absorption spectrum computed with Sc-GW0 exhibits a high oscillator strength between ∼0.5 and 2.0 eV,^52^ unlike our DFT + *U* + *J* approach, it cannot resolve the two distinct transitions at 0.8 and 1.6 eV reported in experimental optical spectra.^27,36,37,41,45^

The experimental presence of the 0.8-eV optical transition and discrepancies in the band gap energies computed with various approaches has led to debate over defining the optical band gap of Co_3_O_4_: some report it as 0.8 eV,^27,39,53^ while others define it to be 1.6 eV.^41,54^ The incongruity in reported band gap energies is scrutinized by Smart, *et al.* in their work modelling Co_3_O_4_ optical transitions with DFT + *U* and a hybrid functional that includes a fraction of Hartree–Fock exchange.^55^ They propose the 0.8-eV optical transition arises from photoinduced formation of a small hole polaron, and that this transition becomes most apparent upon application of uniaxial lattice strain.^55^ Our calculation of the ground-state electronic structure of Co_3_O_4_ using a Hubbard- and Hund- corrected plane-wave pseudopotential approach demonstrates that the low-energy transition at 0.8 eV (and its 0.9-eV shoulder) originate from localized Co^2+^ T_d_ valence bands (e). Additionally, the corresponding empty Co^2+^ T_d_ conduction bands (t_2_) participate in the observed transition at 1.64 eV, indicating the importance of T_d_ Co^2+^ electronic character to this transition as well. Although the computational approach used here is a single-particle method that neglects many-body perturbations and nuclear motion, its ability to capture all the features observed in the experimental dielectric spectrum supports its accuracy in describing the nature of the bands that contribute to optical transitions in Co_3_O_4_. Because the dielectric spectrum reported here is calculated within the Born–Oppenheimer approximation, transitions originating from ground states containing nuclear displacements are not captured, contrasting the description of the 0.8 eV transition as involving a lattice strain-induced small hole polaron by Smart, *et al.*^55^

### Optical phonon enhancement in Co_3_O_4_

**Fig. 3:**
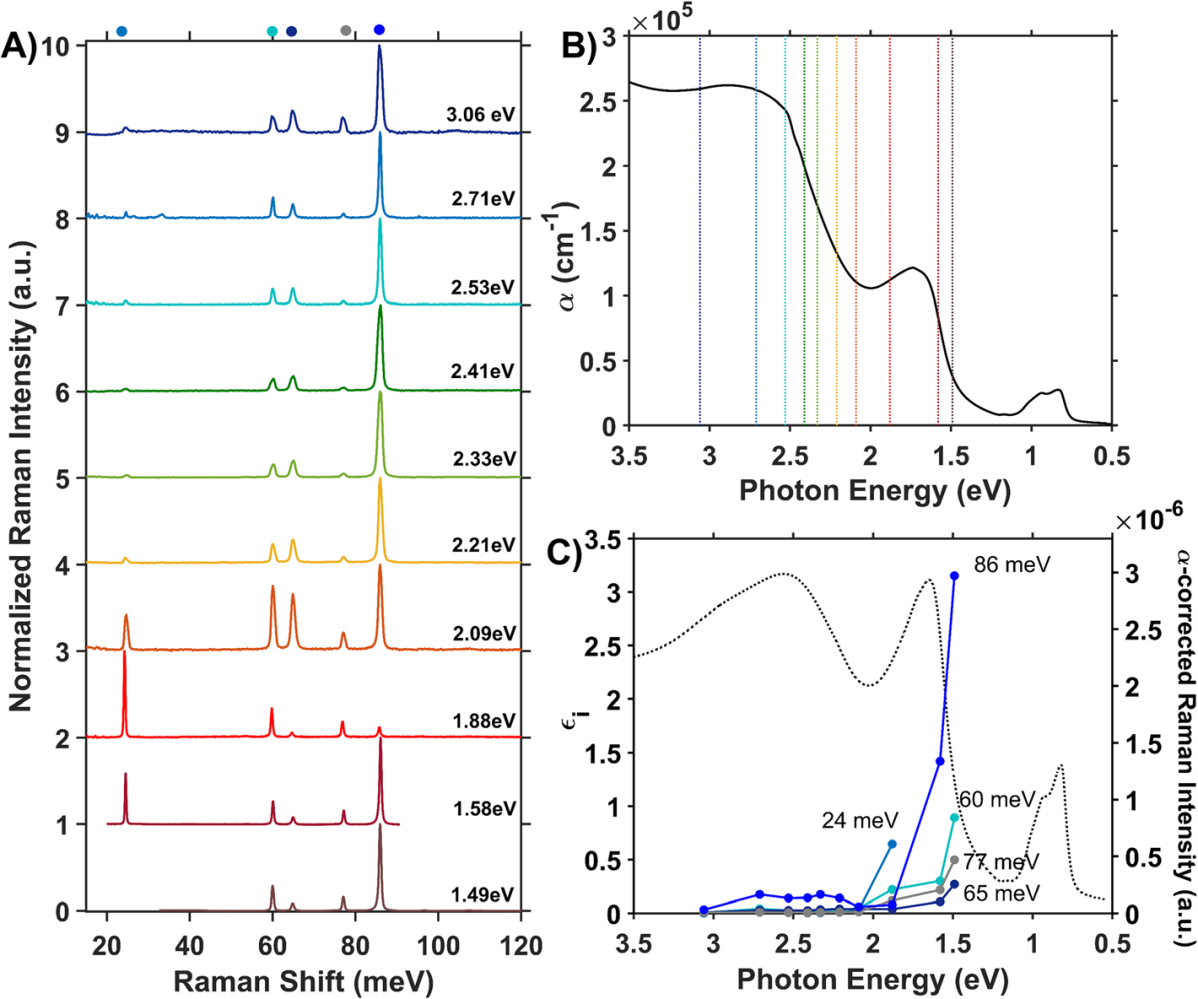
(A) Internally normalized Stokes Raman spectra of Co_3_O_4_ collected with various excitation energies that span the absorption spectrum plotted in (B). Raman spectra are vertically offset for clarity. Because the spectra are internally normalized, only relative changes in phonon mode intensity can be assessed as a function of excitation energy. Note that the spectrum excited with 1.49 eV begins at a Raman shift of 30 meV due to instrumental constraints, primarily the bandwidth of the filter used to remove Rayleigh scattering. (C) Plot of the intensities of the various Raman modes shown in part A corrected for scattering cross section and sample absorption superimposed on the imaginary dielectric spectrum. Note the corresponding color point of each phonon mode indicated above the relevant Raman peak in A. For the full correction, see ESI Fig. S5.†

**Fig. 4:**
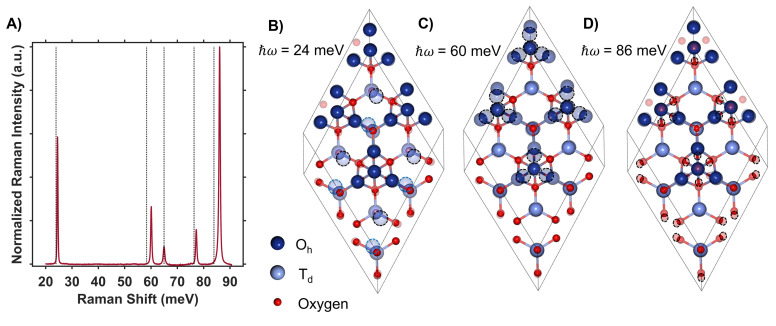
(A) Raman spectrum of Co_3_O_4_ collected with a excitation photon energy of 1.58 eV overlaid with the energies of DFT + *U* + *J*-computed Raman-active phonon modes. The atomic displacements corresponding to the modes at 24 meV (B), 60 meV (C) and 86 meV (D) are shown. The circled atoms demonstrate the vector displacements of the phonon modes in a 2 × 2 × 1 super cell, where T_d_ sites are most shifted at 24 meV, O_h_ sites at 60 meV and oxygens surrounding T_d_ sites at 86 meV. Note that the extra oxygen atoms in the 24 meV and 86 meV vector-displacement images appear from the displacement of the neighboring super-cell.

We assess phonon coupling to various optical transitions in Co_3_O_4_ using Stokes resonance Raman spectroscopy. Fig. 3A plots a series of resonance Raman spectra collected for a 428-nm thick Co_3_O_4_ film deposited on a sapphire substrate using a variety of excitation lasers with photon energies ranging from 1.49 to 3.06 eV, which spans the Co_3_O_4_ absorption spectrum (Fig. 3B). These Raman spectra of Co_3_O_4_ each contain five phonon modes, consistent with previous reports.^56,57^ The phonon mode at 86 meV (oxygen breathing about T_d_ Co^2+^, Fig. 4D) is the most intense at all excitation energies, except *hν*_exc_ = 1.88 eV, which corresponds to the inter-sublattice charge transfer transition (O_h_ Co^3+^ t_2g_ → T_d_ Co^2+^ t_2_). When Co_3_O_4_ is excited with a photon energy of 1.58 eV, which correspond to the lower-energy edge of this inter-sublattice charge transfer peak, the 86 meV phonon mode becomes most intense again. Fig. 3C plots the excitation spectrum for each phonon mode corrected for scattering cross section and sample absorption, which enables the comparison of phonon mode intensities across different excitation energies. This quantitative analysis of relative intensities reveals that there is amplified resonance enhancement of all modes upon excitation at 1.49 eV, indicating strong phonon coupling to this optical transition. This analysis was repeated on a thin film of Co_3_O_4_ deposited on quartz, and the same trends are apparent (Fig. S7†). Given that the 86-meV phonon mode is most intense at an excitation energy of 1.49 eV, we propose it is this phonon mode that most strongly couples to the optical transition at 1.49 eV.

DFT + *U* + *J* was used to calculate the displacement vectors of all 39 optical phonon modes in Co_3_O_4_ at *k*-point *Γ* (Table S2†). White and DeAngelis determined through evaluating the Raman selection rules that there are five Raman active modes in normal spinel oxides.^58^ The symmetry of these modes was compared with the displacement vectors computed with DFT + *U* + *J* to identify the Raman active modes, the energies of which are overlaid with the Raman spectrum in Fig. 4A. From the resonance Raman profile (Fig. 3A and C), the phonon modes with energies of 24, 60, and 86 meV exhibit the most significant resonance enhancement upon excitation at 1.49 eV, corresponding to the onset of the absorption feature centered at 1.64 eV. These modes correspond to motion of tetrahedral Co^2+^ (24 meV), octahedral Co^3+^ (60 meV), and oxygen stretching symmetrically about Co^2+^ T_d_ atoms (86 meV, Fig. 4B–D).

The observed enhancement of the 24-meV (T_d_ Co^2+^motion) and 60-meV (O_h_ Co^3+^ motion) phonons at a Raman excitation energy of 1.49 eV is related to the contribution of both T_d_ Co^2+^ and O_h_ Co^3+^ electronic character to this optical transition, which corresponds to population of empty T_d_ Co^2+^ t_2_ conduction band states from O_h_ Co^3+^ t_2g_ valence band states. Although both T_d_ Co^2+^ and O_h_ Co^3+^ phonon modes exhibit resonance enhancement because of associated electronic character in the optical transition centered at 1.64 eV, it is the oxygen breathing mode about Co^2+^ T_d_ ions at 86 meV that exhibits the most enhancement. The degree of energetic overlap between the O 2p and Co^2+^ T_d_ projected density of states in the conduction band at the energy of the O_h_ Co^3+^ t_2g_ →T_d_ Co^2+^ t_2_ transition is significant (Fig. 2B and D) and supports the observed enhancement of the 86-meV phonon, which corresponds to motion of oxygen atoms along their bond axes to T_d_ Co^2+^.

### Temperature dependence of optical transitions

**Fig. 5:**
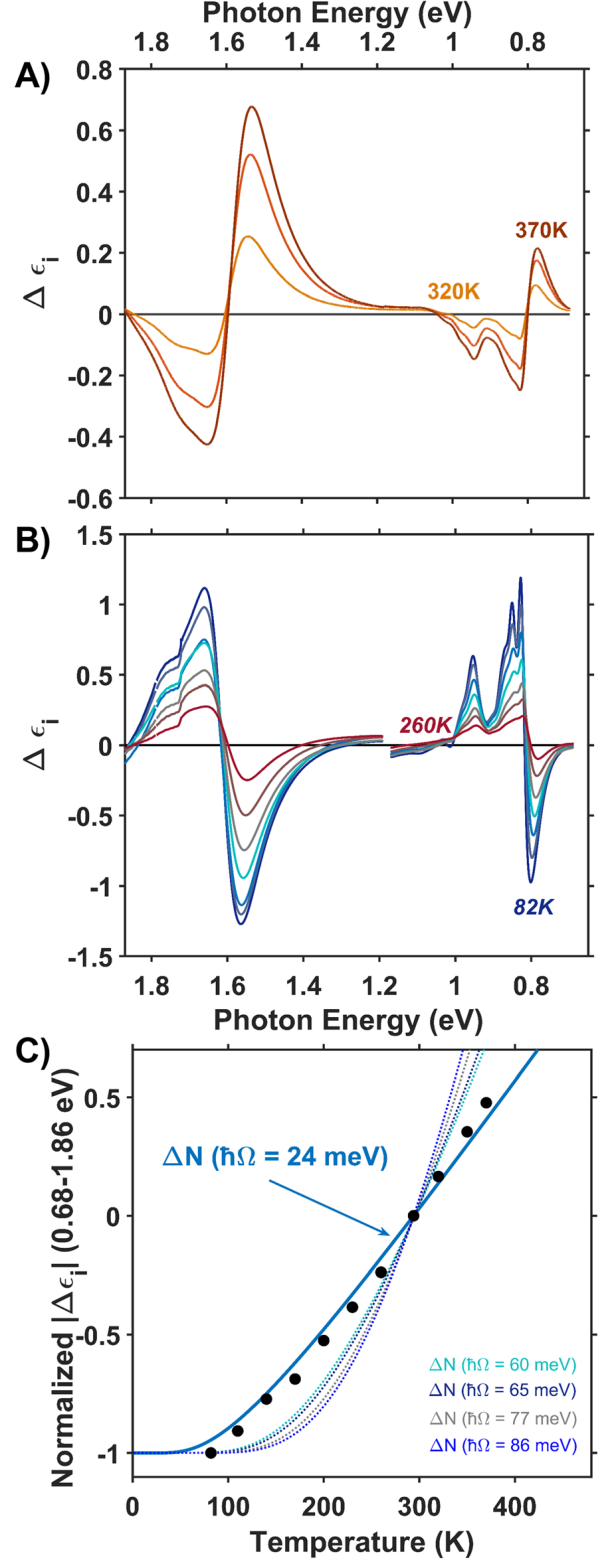
Thermal difference imaginary dielectric spectra of Co_3_O_4_ collected at temperatures above (A) and below (B) room temperature (294 K). (C) Absolute value of the thermal difference spectra integrated from 0.68 to 1.86 eV and normalized to the integrated intensity of the spectrum collected at 82 K plotted *versus* temperature (black circles). The solid blue line plots the temperature-dependent change in Bose–Einstein population of a phonon with an energy of 24 meV. Bose–Einstein distributions corresponding to the energies of the other Raman active phonon modes are plotted as dotted lines.

To further understand the role of phonon coupling in the optical transitions of Co_3_O_4_, we measured the dependence of its dielectric spectrum on temperature between 82 and 470 K. Fig. 5A and B plot the resulting thermal difference spectra (TDS) calculated according to eqn (1).

1 Δ*ε*_i_(*T*) = *ε*_i,T_ − *ε*_i,294K_

We assessed the impact of temperature on the dielectric spectrum by integrating the intensity of the thermal difference spectra. Fig. 5C (blue) plots the absolute value of the thermal difference spectra integrated from 0.68 to 1.86 eV and normalized to the integrated intensity obtained from the spectrum collected at 82 K. To account for sign changes, the integrands of spectra collected below room temperature are shown as negative, and those collected above room temperature are positive. For phonon-coupled optical transitions, we expect the change in intensity with changing temperature to be proportional to the change in population of the coupled phonons. The thermal population of phonon modes is dictated by the Bose–Einstein distribution shown in eqn (2), where *ħ*Ω is the phonon energy. Normalization of the differential spectra to an arbitrary temperature, in this case 82 K, leads to eqn (3), where we compare the change in intensity of the dielectric spectrum (Δ*ε* defined in eqn (1)) to the change in population of the Raman active phonon modes at various temperatures.2
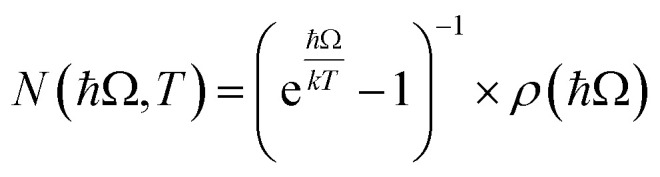
3
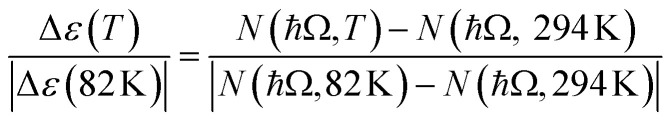


The temperature dependence of the TDS intensity overlays well with the change in population of the 24-meV Raman-active phonon with temperature predicted by the Bose–Einstein distribution (eqn (3)). This agreement suggests that the low energy optical transitions (1.64 eV, 0.96 eV and 0.82 eV) are coupled strongly to the thermal population of this phonon. Interestingly, the 24-meV phonon mode is not the most enhanced in resonance Raman spectra collected with an excitation photon energy of 1.49 eV; however, both the 24-meV and 86-meV phonon modes are described by displacement vectors primarily of or around T_d_ Co^2+^ions (Fig. 4B and D). Evidence of thermally activated optical transitions combined with strong phonon coupling to optical transitions observed in resonance Raman spectra (*vide supra*) suggests an optically accessed polaronic state related to T_d_ Co^2+^. Compared to resonance Raman measurements, thermal difference spectra are not as precise in determining the energies of the specific phonon modes coupling to the optical transition, as there may be multiple phonon modes contributing to thermal activation. However, the Bose–Einstein distribution corresponding to a phonon energy of 24 meV overlays the temperature-dependent TDS intensities much better than the distributions corresponding to other Raman-active phonon modes (Fig. 5C). We therefore interpret the threshold phonon energy of 24 meV to be where thermal activation occurs. Similar temperature-dependent behavior is observed in α-Fe_2_O_3_: at the excitation energy where maximal Raman enhancement is observed (∼2.2 eV), the strongest temperature-dependence in the optical spectrum is also present.^29^ Thermal activation of optical transitions in α-Fe_2_O_3_ is the proposed mechanism by which direct excitation into intrinsic polaronic states occurs.^29,30^ The similarities in the trends of Raman enhancement and the temperature-dependence of the dielectric spectrum observed for Co_3_O_4_ and α-Fe_2_O_3_ support the presence of an optically accessible polaronic state in Co_3_O_4_ arising from coupling to intrinsic, dynamic lattice distortions (phonons) (Fig. 1, red arrow).

### Co_3_O_4_ compared to ZnCo_2_O_4_: assessing the role of T_d_ Co in spinel oxides

**Fig. 6:**
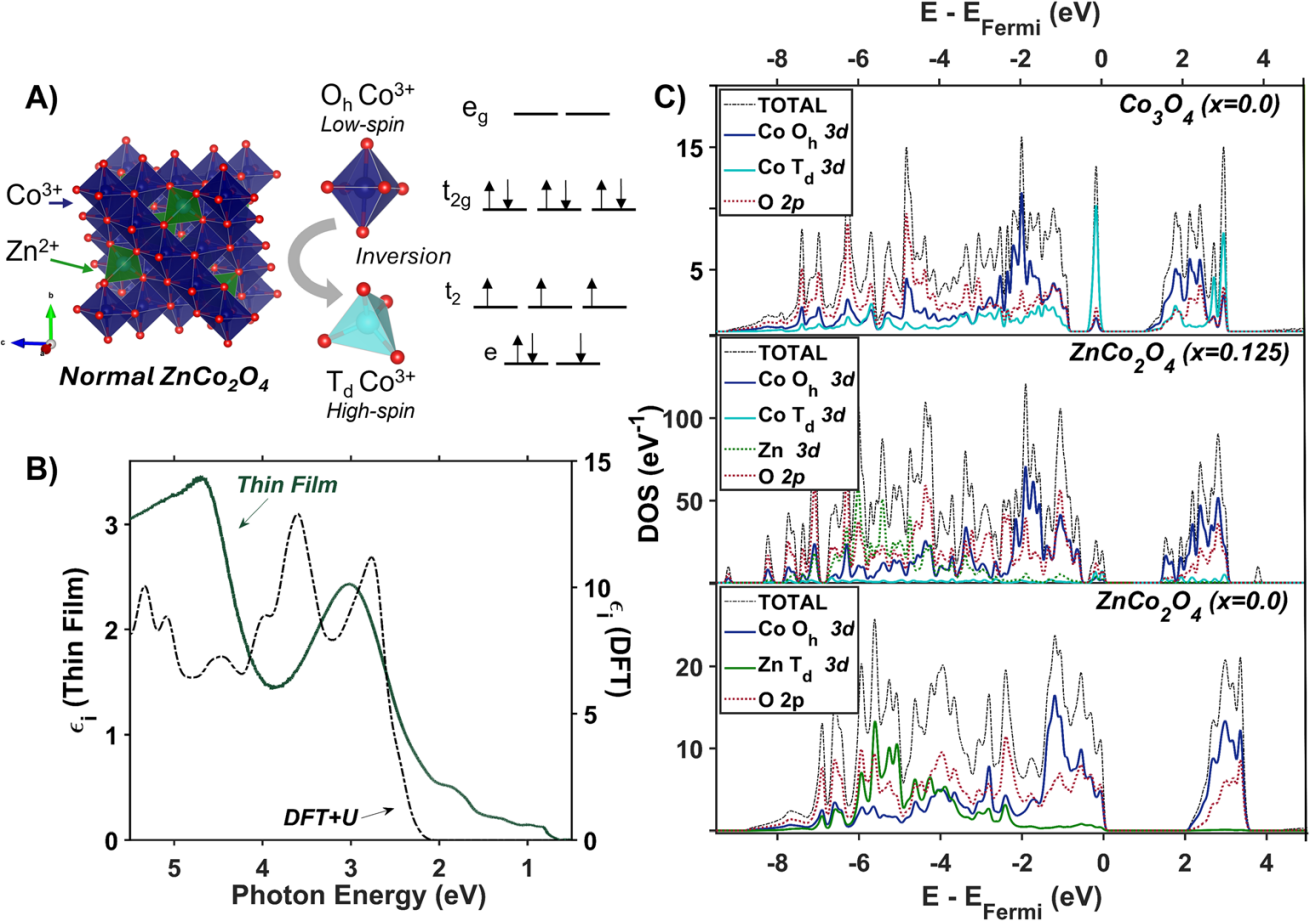
(A) Unit cell of normal ZnCo_2_O_4_ with associated crystal field splitting diagrams of Co^3+^ in octahedral and tetrahedral coordination. (B) Plot of experimentally (solid green line) and computationally (dashed black line) determined imaginary dielectric spectra of ZnCo_2_O_4_. The computed dielectric spectrum was calculated for normal ZnCo_2_O_4_. (C) The electronic density of states calculated with Hubbard-corrected DFT for normal Co_3_O_4_ (top), ZnCo_2_O_4_ with an inversion factor of 0.125 (middle), and normal ZnCo_2_O_4_ (bottom).

The resonance Raman and thermal difference spectra of Co_3_O_4_ suggest that Co^2+^ ions occupying T_d_ sites are involved in the phonon-coupled optical transitions. To further understand the role of T_d_ Co^2+^ in the optical spectra of Co_3_O_4_, we assessed the optical properties of ZnCo_2_O_4_, where Zn^2+^ replaces T_d_ Co^2+^ (Fig. 6A). We computed the imaginary dielectric spectrum for normal ZnCo_2_O_4_ with DFT + *U* (see ESI for details†). Within normal ZnCo_2_O_4_, the T_d_ Zn^2+^ 3d orbitals are completely filled, and the O_h_ Co^3+^ 3d orbitals have a low-spin configuration, termed “quasi close-shelled”.^59,60^ With no unpaired electrons present, the Hund parameter, *J*, is not implemented in these calculations. The DFT + *U* calculations of normal ZnCo_2_O_4_ reveal two absorption bands at and above ∼2.5 eV, while the experimental spectrum only has one transition in this region. Literature reports assign the experimentally observed feature at 3.0 eV to an LMCT-type transition,^27,38^ which matches the lower energy peak (2.5 eV) observed in the DFT + *U* dielectric spectrum. The 3.5 eV peak in the DFT + *U* dielectric spectrum computed here is associated with an intra-sublattice charge transfer (O_h_ Co^3+^ t_2g_ → O_h_ Co^3+^ e_g_). Because this is a charge transfer between two different lattice sites, the Laporte selection rule does not apply. Due to the hybridized nature of the Co 3d and O 2p orbitals in both the valence and conduction bands, we assign the experimental dielectric peak at 3.0 eV to a combination of LMCT and intra-sublattice charge transfer transitions (see Fig. S11 in the ESI†). Interestingly, when comparing the experimental peak shape of the dielectric transition at 3.0 eV in ZnCo_2_O_4_ (Fig. 6B) to the analogous transition in Co_3_O_4_ (Fig. 2C and S12†), it becomes apparent that the shoulder in Co_3_O_4_ (labeled e in Fig. 2C) is at the same energy as the peak center of the experimental ZnCo_2_O_4_ transition. This energetic alignment in the experimental dielectric spectra, used to assign the 2.90-eV shoulder in Co_3_O_4_ as excitation into O_h_ Co^3+^ e_g_ bands, has been observed previously in a Zn dopant study of Co_3_O_4_.^38^

The lower-energy region of the experimental dielectric spectrum of ZnCo_2_O_4_ contains broad and weak features at 1.79, 0.98 and 0.82 eV, which coincide with where Co_3_O_4_ has transitions associated with Co^2+^ T_d_. X-ray fluorescence analysis indicates the stoichiometry of the ZnCo_2_O_4_ film used to produce the dielectric spectrum is 1.94 ± 0.04 Co : Zn, and the powder X-ray diffraction pattern indicates the film is phase-pure spinel (Fig. S3†). We therefore suspected that our ZnCo_2_O_4_ film may be slightly inverted and contain a small population of cobalt in tetrahedral sites, consistent with previous reports.^61^ In an effort to control cation distribution in ZnCo_2_O_4_, we changed the temperature at which ZnCo_2_O_4_ films were annealed following spin-coating. We observe that annealing above 600 °C induces phase separation as features associated with ZnO become apparent in the X-ray diffraction pattern and Raman spectra (Fig. S13 and S14†). Additionally, as annealing temperature increases, the low energy peaks observed in the dielectric spectrum increase in intensity (Fig. S15†). We therefore suspect that the films containing ZnO also contain Co_3_O_4_, which accounts for the presence of the intense low-energy peaks in the dielectric spectrum (Fig. S15†). With no ZnO peaks present in X-ray diffraction or ZnO phonon modes observed in Raman for the films annealed at 600 °C, we hypothesize that the weak, low-intensity features observed in the dielectric spectra of these films arise from cation inversion, whereby a fraction of the Co ions occupy T_d_ sites and a fraction of the Zn ions occupy O_h_ sites. We describe the Co ions in T_d_ sites as substitutional lattice defects.

To investigate the impact of cation inversion on the optical spectra of ZnCo_2_O_4_, we computed the electronic structure of inverted ZnCo_2_O_4_ using DFT + *U* + *J* (see ESI for details†). Inverted ZnCo_2_O_4_ (i-ZnCo_2_O_4_) was modeled by switching two O_h_ Co^3+^ ions with two T_d_ Zn^2+^ ions within a 2×2×2 supercell of normal ZnCo_2_O_4_ to produce an inversion factor of *x* = 0.125 while maintaining charge balance and stoichiometry. The resulting two T_d_ Co^3+^ ions were modeled to be antiferromagnetically coupled to maintain net-zero magnetization. Compared to normal ZnCo_2_O_4_ (n-ZnCo_2_O_4_), i-ZnCo_2_O_4_ contains an isolated state at the valence band-edge with primarily T_d_ Co and O 2p character (Fig. 6C, middle and bottom). The conduction band edge in i-ZnCo_2_O_4_ appears at a lower energy than that in n-ZnCo_2_O_4_ (similar to Co_3_O_4_), and i-ZnCo_2_O_4_ contains an isolated region at the conduction band-edge of primarily O_h_ Co and O 2p character (Fig. 6C, middle and top). The band-edge character of i-ZnCo_2_O_4_, despite the 3+ oxidation state of the cobalt ion in T_d_ sites, has more similarities to that of Co_3_O_4_ than n-ZnCo_2_O_4_ (Fig. 6C). Thus, the presence of Co (T_d_) in spinel oxides results in low-energy d-to-d transitions, regardless of its oxidation state (+2 or +3). Because there is no evidence of phase separation (*i.e.* presence of Co_3_O_4_) to describe the low-energy features in the dielectric, we conclude the synthesized ZnCo_2_O_4_ contains at least a small degree of inversion.

**Fig. 7:**
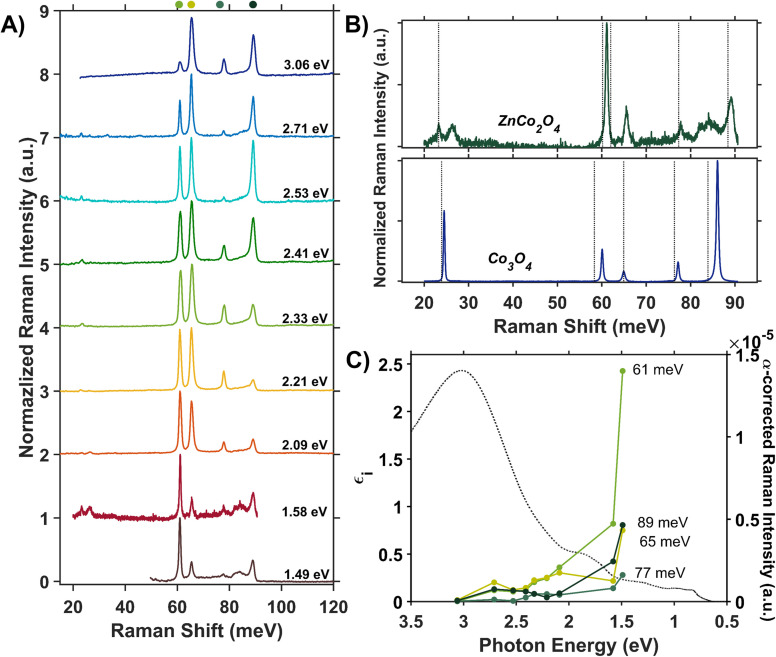
(A) Internally normalized Raman spectra of a ZnCo_2_O_4_ thin film deposited on a sapphire substrate collected with excitation photon energies that span its absorption spectrum. Because the spectra are internally normalized, only relative changes in phonon mode intensity can be assessed as a function of excitation energy (see ESI† for full work-up of Raman data). (B) Raman spectra of ZnCo_2_O_4_ (top) and Co_3_O_4_ (bottom) excited at a photon energy of 1.58 eV are overlaid with the Raman-active phonon modes computed for normal ZnCo_2_O_4_ and Co_3_O_4_, respectively. (C) Plot of the intensities of the various Raman modes shown in part A corrected for scattering cross section and sample absorption superimposed on the imaginary dielectric spectrum of ZnCo_2_O_4_. Note the corresponding color point of each phonon mode indicated above the relevant Raman peak in A.

The Raman spectrum of ZnCo_2_O_4_ has five distinct modes (Fig. 7A and B) consistent with previous reports.^62,63^ The phonon mode frequencies are similar to those observed in Co_3_O_4_, which is expected, as both materials adopt the spinel crystal structure. Additionally, the 23-meV, 61-meV, and 89-meV phonon modes in ZnCo_2_O_4_ are described by the same phonon motions as in Co_3_O_4_: motion of T_d_ metal center motion of O_h_ metal center, and oxygen stretching about T_d_ sites respectively (Fig. 4B–D). Fig. 7B (top) plots the experimental Raman spectrum of ZnCo_2_O_4_ overlaid with the computed phonon modes of normal ZnCo_2_O_4_. While the five prominent phonon modes are accounted for by DFT + *U* phonon calculations, there are two broad and weak features at 26 meV and 85 meV that do not appear in calculations. Both phonons have similar energies to corresponding modes observed in Co_3_O_4_, which are both related predominantly to T_d_ sites (Fig. 7B, bottom). The discrepancy between the energies of experimentally observed Raman-active optical phonon modes and the computed modes of normal ZnCo_2_O_4_ can be explained by the sample crystallizing with a degree of inversion. With a small percentage of tetrahedral sites occupied by Co^3+^, and the rest by Zn^2+^, the phonon modes dominated by tetrahedral motion (∼24 meV and ∼89 meV) split into two distinct distributions. This phenomenon has been observed in other inverted spinel oxides^64^ and is further evidence of the ZnCo_2_O_4_ films crystallizing with a small percent occupation of cobalt in tetrahedral sites (Fig. 7B).

Unlike Co_3_O_4_, the resonance Raman profile of ZnCo_2_O_4_ does not exhibit increased resonance enhancement of the phonons associated with T_d_ motion (23 and 89 meV) with decreasing excitation energy. In contrast, the 61-meV phonon, related to Co O_h_ motion (described by Fig. 4B), exhibits enhanced intensity in ZnCo_2_O_4_ relative to all the other modes as excitation photon energy decreases (Fig. 7A). Interestingly, when the Raman profile is corrected for scattering cross section and sample absorption, the 61-meV mode is most enhanced at an excitation photon energy of 1.49 eV – the same excitation photon energy at which the most significant resonance enhancement is observed in Co_3_O_4_ (Fig. 3C and Fig. 7C). This analysis was repeated on a thin film of ZnCo_2_O_4_ deposited on quartz, and the same trends are apparent (Fig. S8†). The similarity in excitation photon energy where resonance enhancement is observed in ZnCo_2_O_4_ and Co_3_O_4_ indicates that the onset of the optical transition centered at ∼1.6 eV is significant for both materials.

Based on the calculated density of states of inverted ZnCo_2_O_4_ (Fig. 6C), the experimental optical transition observed at 1.6 eV is assigned to metal-to-metal charge transfer (MMCT) from bands with T_d_ Co^3+^ character (arising from the T_d_ Co substitutional defects) to bands with O_h_ Co^3+^ character. The most enhanced Raman spectrum (*hν*_exc_ = 1.49 eV) occurs upon excitation at the onset of the 1.6-eV transition, implying that the presence of the Co^3+^ T_d_ substitutional defect is crucial for the observed enhancement of the O_h_ phonon in ZnCo_2_O_4_. The conduction band character associated with the 1.6-eV transition arises from 3d orbitals associated with Co^3+^ O_h_, the same site associated with the main vibrational motion of the 61-meV phonon (Fig. 4C). Although the strong resonance enhancement observed for the 61-meV phonon in ZnCo_2_O_4_ is evidence of strong coupling between this mode and the optical transition at 1.6 eV, we do not observe significant temperature dependence of this transition in thermal difference spectra when compared to Co_3_O_4_ (see Fig. S16 in ESI†). In fact, unlike Co_3_O_4_, the thermal difference spectra collected for ZnCo_2_O_4_ at elevated temperatures do not exhibit any well-defined features corresponding to spectral features observed in the dielectric spectrum at room temperature.

The spectral and computational results reported here for ZnCo_2_O_4_ and Co_3_O_4_ indicate that Co 3d character at the band edge mediates phonon-coupled optical transitions (Fig. 3C, 4C, D and 7C). However, the contrast in temperature-dependence of the optical transition where both materials exhibit the strongest phonon coupling (1.6 eV) suggests fundamentally different processes dictating the observed enhancement. We interpret our findings in Co_3_O_4_ as direct population of an intrinsic polaronic state *via* photoexcitation, which is strongly influenced by the contributions of Co^2+^ T_d_ ions to band-edge states. In ZnCo_2_O_4_, we interpret the experimental ZnCo_2_O_4_ sample to have a small percent occupation of tetrahedral sites by Co based on the presence of low-energy optical transitions associated with T_d_ Co. Although the occupation of Co^3+^ in tetrahedral sites enables the optical transition and phonon enhancement at 1.6 eV, the lack of significant temperature dependence of this optical transition indicates that the phonon-coupled optical transitions leading to Raman enhancement in ZnCo_2_O_4_ are fundamentally different from those observed in Co_3_O_4_.

We suspect that the differences observed in the thermal difference spectra of Co_3_O_4_ and ZnCo_2_O_4_ are related to the disruption in the translational symmetry of the lattice induced by cation inversion in ZnCo_2_O_4_. From assignment of optical transitions in Co_3_O_4_, the low-energy optical transitions are either highly localized transitions between neighboring Co atoms or intra-atomic transitions in T_d_ Co (Table 1). With fewer Co atoms in T_d_ sites, these optical transitions become suppressed, as observed in ZnCo_2_O_4_ (Fig. 6B); however, the localized transitions still exhibit phonon coupling, leading to the observed resonance Raman enhancements (Fig. 7C). The lack of temperature dependence of these transitions in ZnCo_2_O_4_ suggests that, although these transitions access localized phonon-coupled states, the localization is related to the static lattice defect of tetrahedral cobalt rather than thermally induced dynamic lattice displacements within a pristine, translationally symmetric lattice. The lack of thermal dependence in ZnCo_2_O_4_, but presence of resonance Raman enhancement of O_h_ phonon modes upon excitation of a transition from T_d_ Co to O_h_ Co, is strong evidence of an optically accessed polaronic state. We therefore conclude that in both Co_3_O_4_ and ZnCo_2_O_4_ there is evidence of an optically accessible polaronic state. However, in Co_3_O_4_ formation of the polaronic state is due to intrinsic, dynamic lattice deformations caused by thermally activated phonons (exactly analogous to the mechanism we observe in hematite), whereas in ZnCo_2_O_4_, photoinduced polaron formation is mediated by static lattice defects arising from cation inversion.

## Conclusions

The optical characterization of Co_3_O_4_ and ZnCo_2_O_4_ reported here shows that low energy transitions at 0.8, 0.9 eV and 1.6 eV arise from tetrahedrally coordinated cobalt ions, regardless of oxidation state (Co^2+^, Co^3+^). The d–d transition observed at 1.6 eV in both Co_3_O_4_ and ZnCo_2_O_4_ is a phonon-coupled optical transition. In Co_3_O_4_, the combination of temperature-dependent intensity and resonance Raman enhancement at the onset of the O_h_ Co^3+^ → T_d_ Co^2+^ optical transition (1.64 eV) is evidence that this transition directly populates an intrinsic polaron state coupled to thermally activated phonons. Although the resonance Raman profile of ZnCo_2_O_4_ exhibits a similar Raman enhancement upon excitation of the 1.6-eV transition, the observed inversion and lack of temperature dependence suggests that this photoexcited polaron forms due to the presence of tetrahedral cobalt substitutional defects within the lattice. We conclude that T_d_-coordinated cobalt is a significant factor in phonon-coupled transitions for cobalt-containing spinel oxides. The contrast in the mechanism of polaron formation (intrinsic or self-trapped polaron *vs.* defect-mediated polaron) observed for these similar materials is an important insight that we anticipate will help uncover mechanisms of photoinduced polaron formation in other oxide materials. Although both mechanisms of polaron formation observed here have been previously reported, understanding the spectral signatures that distinguish them is crucial for further development of oxide materials for photo-applications.

## Data availability

Data supporting this article have been included as part of the ESI.† Primary data are available from the authors upon reasonable request.

## Author contributions

E. P. C., J. L. S., and K. E. K. contributed to conceptualization of the project and interpretation of the data; K. E. K. supervised the project. E. P. C. performed most experiments and computations. J. L. S. contributed to some computations for Co_3_O_4_. M. T. R. collected low-frequency Raman spectra. E. P. C. wrote the manuscript. All authors reviewed the manuscript.

## Conflicts of interest

There are no conflicts to declare.

## Supplementary Material

SC-OLF-D5SC01909E-s001
