# Supplementary material for: Local coordination geometry within cobalt spinel oxides mediates photoinduced polaron formation
Source: Chem Sci. 2025 May 12;16(24):10759–70. doi: 10.1039/d5sc01909e (PMC12089999; doi:10.1039/d5sc01909e)
Supplement: SC-016-D5SC01909E-s001 [file SC-016-D5SC01909E-s001.pdf]

Supplementary Information (SI) for Chemical Science.
This journal is © The Royal Society of Chemistry 2025

Supporting Information

Local Coordination Geometry within Cobalt Spinel Oxides Mediates Photoinduced Polaron Formation

Erica P. Craddock,[†] Jacob L. Shelton,[†,‡] Michael Ruggiero,[†] Kathryn E. Knowles[†]*

[†]*Department of Chemistry, University of Rochester, Rochester NY 14627, USA*

*corresponding author e-mail: kknowles@ur.rochester.edu

I. Experimental Methods

Sample Preparation – Thin films of Co_3O_4 and $ZnCo_2O_4$ were prepared using metal nitrate salts as precursors: for Co_3O_4, 3 mmol of Co(II) nitrate hexahydrate and for $ZnCo_2O_4$, 2 mmol of Co(II) nitrate hexahydrate and 1 mmol of Zn(II) nitrate tetrahydrate. These nitrate salts were combined with 4.5 mmol citric acid monohydrate and 4.5 mmol ethylene glycol in 6 mL of ethanol. Aliquots (50 μL) of precursor solutions were spin-coated at 5000 rpm onto sapphire substrates and annealed at 600 °C for 5 minutes. Multiple layers were deposited in this manner before a final annealing at 600 °C for 24 hours. One annealed aliquot contributes ~40 nm to film thickness. To determine the thickness of the films, samples were etched with a razor blade and thickness was determined by etch profile using atomic force microscopy (AFM) for films <100 nm (NTMDT AFM Microscope, Asylum MFP-3D AFM Microscope) and an Ambios XP-200 Surface Profiler for films >100 nm. Films <100 nm were used to collect optical functions and thicker films (>100 nm) were used to collect Raman spectra.

Structural Analysis – Powder X-ray diffraction (XRD) patterns were collected with a 0.6-mm slit at low grazing Cu K-alpha emission angles using a Bruker D8 Advance Diffractometer. Energy dispersive X-ray emission spectra of each sample were collected in triplicate under vacuum with 3 mm beam collimation using Shimadzu EDX-8100. Thin samples used for optical functions and thick samples used for Raman were all verified as phase-pure spinels by XRD and the ratio of Co:Zn was determined by X-ray emission (Figures S1-S4).

Optical Measurements - An Agilent Cary 7000 UMA Spectrophotometer was used to collect transmission and reflection spectra of all films (2640-190 nm). These spectra were processed in MATLAB to extract the complex dielectric functions using previously reported methods.[1,2] Temperature-dependent measurements were collected with the same spectrophotometer using either APD Cryogenics Inc. Model LT-3-110 Heli-Tran cryostat system run at 10^{-5} Torr vacuum with a turbo molecular vacuum pump (77-294 K) or using thermal ceramic heaters and an Omega Platinum Series temperature benchtop controller

[‡]current address: National Renewable Energy Laboratory, Chemistry and Nanoscience Center, Golden, CO, 80401, USA

(294-370K). Raman spectra were collected using CW diode lasers with wavelength emissions of 830 nm (1.49 eV), 785 nm(1.58 eV), 660 nm (1.88 eV), 594 nm (2.09 eV), 561 nm (2.21 eV), 532 nm (2.33 eV), 515 nm (2.41 eV), 491 nm (2.53 eV), 457 nm (2.71 eV) and 405 nm (3.06 eV) and Princeton Instruments Monochromators equipped with a CCD detector. Laser power was kept < 30 mW to limit the possibility of sample heating. The sample Raman data was corrected with cyclohexane spectra for both Raman shift and scattering cross section (Figures S5-S6), and the sample's thickness was used to correct for the sample's absorption coefficient at each wavelength.

Low-frequency Raman spectroscopy experiments were performed using a 785-nm excitation source coupled with commercial Raman probe module from Coherent. The excitation and scattered radiation were collected using a Nikon LV100 microscope that utilized a 50x polarization-conserving objective, which resulted in a spot size of ca. 4 micrometers. The scattered radiation was dispersed using an Andor Shamrock 500i spectrograph, which consisted of a 1200 l/mm grating blazed at 750 nm, and the radiation was detected using an Andor iDus 416 CCD camera.

II. Computational Methods

Density functional theory (DFT) and Hubbard-corrected DFT were performed using the pseudopotential plane wave package implemented in Quantum ESPRESSO.[3–5] High plane wave cut-off energies (1200 eV for Co_3O_4 and 1100 eV for normal and inverted $ZnCo_2O_4$) were used to ensure the total energy and total interatomic forces were converged to 10 meV and 0.10 meV/Å, respectively. Both Co_3O_4 and $ZnCo_2O_4$ were sampled over 6x6x6 K-point grids. The Perdew, Burke, and Ernzerh exchange-correlation functional revised for solids (PBEsol)[6,7] and Optimized Norm-Conserving Vanderbilt (ONCV) [8,9] for pseudopotentials were implemented throughout this work. Using the Broyden–Fletcher–Goldfarb–Shanno (BFGS) algorithm,[10–12] iterative geometric relaxations were performed before wavefunctions were calculated to be used for final non-self-consistent field calculations (bands, density of states or dielectric).

Normal Co_3O_4 and $ZnCo_2O_4$: Converged values for plane wave cutoff and k-point grid were determined using the 14-atom primitive cell. Hubbard's *U* (electron correlation correction)[13] and Hund's *J* (local magnetization correction)[14] values were calculated from first principles using a linear response method (Table S1).[13,15] Calculations for Co_3O_4 applied both Hubbard *U* and Hund *J* corrections (DFT+*U*+*J*) due to the high-spin crystal field splitting and antiferromagnetic ordering of T_d Co^{2+} ions (Figure 1A).[16] The T_d Co^{2+} ions in Co_3O_4 were corrected with both *U* and *J* parameters, whereas the O_h Co^{3+} only had a *U* correction due to its low-spin crystal field splitting. Only a Hubbard *U* correction was employed for normal $ZnCo_2O_4$ calculations (DFT+*U*), specifically for O_h Co^{3+}. The electronic bands and projected densities of states were determined by first calculating the self-consistent field (SCF)

wavefunctions with associated Hubbard and Hund corrections and then performing a Fourier interpolation to a larger grid of k-points. Phonon modes of both materials were calculated with density functional perturbation theory (DFPT) using the PHonon code implemented in Quantum ESPRESSO.[3–5] Starting with the same relaxed ground-state configuration used to calculate the electronic states, the dynamical matrices were evaluated over a uniform 4x4x4 grid of q-points.

Inverted $ZnCo_2O_4$*:* Using a 2x2x2 supercell of normal $ZnCo_2O_4$, two Co^{3+} ions traded occupation with two Zn^{2+} ions to produce a net inversion parameter of 0.125 (see Supporting Information Figure S9). The occupation of Co^{3+} in T_d sites necessitated both U and J corrections due to the unpaired electronic configuration (Figure 5A). The Hubbard and Hund-corrected self-consistent field (SCF) wavefunctions were calculated for this supercell and used to calculate the projected density of states.

III. Thin film sample characterization

Thin films of Co_3O_4 and $ZnCo_2O_4$ prepared by spin coating were all characterized by X-ray diffraction, energy dispersive X-ray emission spectroscopy, transmission and reflection optical spectroscopy and profiled for thickness with either profilometry or atomic force microscopy. For $ZnCo_2O_4$ samples, the X-ray emission spectra were used to calculate the Co:Zn ratio by integration of the Kα peak emission of both metals. Figures S1 – 4 show these characterization data for the films of Co_3O_4 and $ZnCo_2O_4$ on sapphire used to collected Raman and optical spectra. The complex dielectric functions were extracted from the transmission and reflection spectra using a previously reported method based on the Fresnel equations.[1,2]

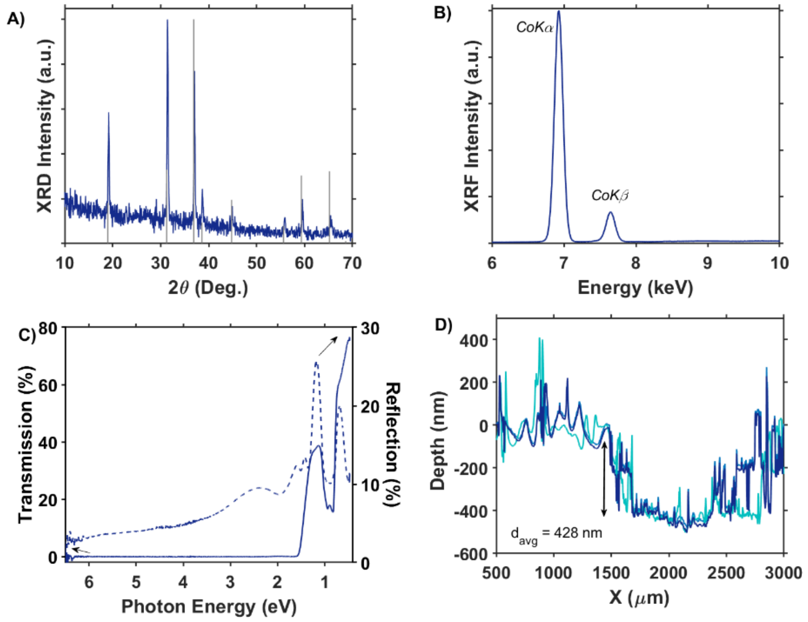

Figure S1. *Characterization of Co_3O_4 film used for Raman collection.* **(A)** X-ray diffraction pattern overlaid with a reference pattern for Co_3O_4 (ICDD 04-016-4508). **(B)** Energy dispersive X-ray emission spectrum, **(C)** optical transmission and reflection spectra and **(D)** depth profile from profilometry (d_{avg} = 428 ±16 nm).

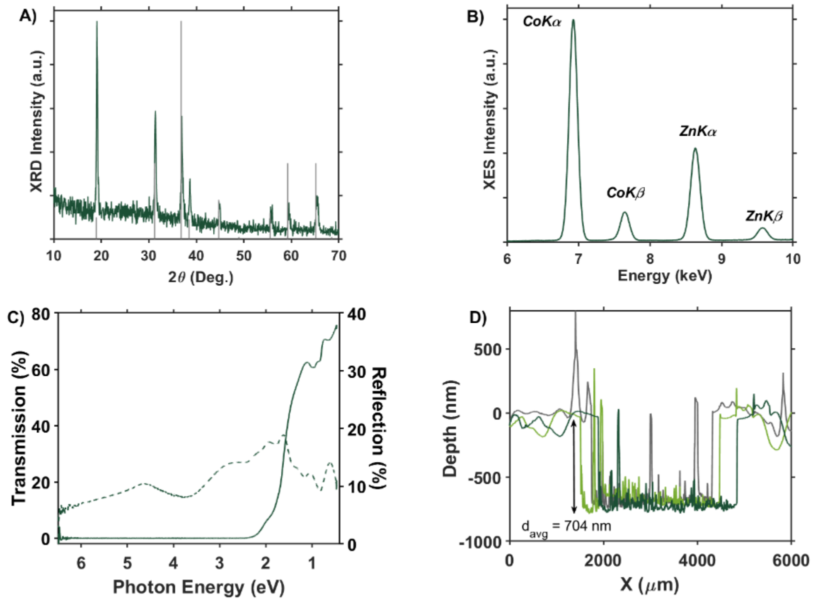

Figure S2. *Characterization of $ZnCo_2O_4$ film used for Raman collection.* **(A)** X-ray diffraction pattern overlaid with a reference pattern for $ZnCo_2O_4$ (ICDD 00-023-1390). **(B)** Energy dispersive X-ray emission spectra; Co:Zn ratio 2.07±0.004 determined from the ratio of integrated areas of the Co Kα and Zn Kα peaks. The uncertainty in the Co:Zn ratio was determined from the standard deviation of three measurements. **(C)** Optical transmission and reflection spectra and **(D)** depth profile from profilometry (d_{avg} = 704 ± 27 nm).

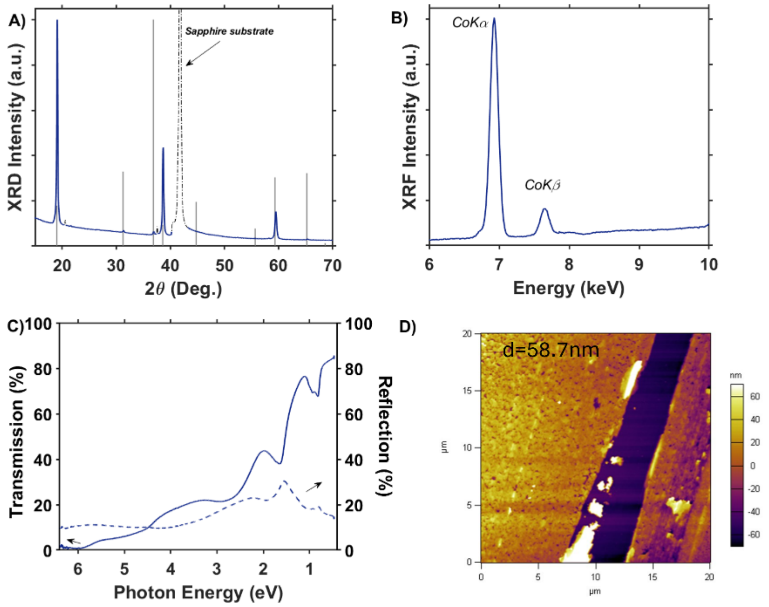

Figure S3. *Characterization of Co_3O_4 film used for optical spectra and temperature-dependent optical spectra*. **(A)** X-ray diffraction pattern overlaid with a reference pattern for Co_3O_4 (ICDD 04-016-4508). **(B)** Energy dispersive X-ray emission spectrum of $ZnCo_2O_4$. **(C)** Transmission and reflection spectra and **(D)** atomic force microscopy image over sample etch (d= 58.7).

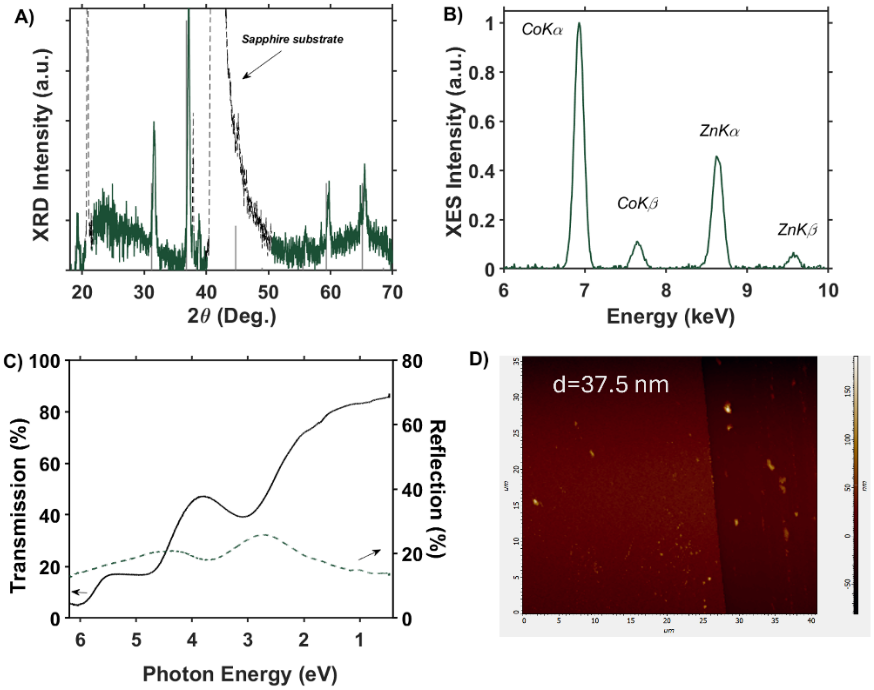

Figure S4. *Characterization of $ZnCo_2O_4$ film used for optical spectra and temperature-dependent optical spectra***. (A)** X-ray diffraction pattern overlaid with a reference pattern for $ZnCo_2O_4$ (ICDD 00-023-1390). **(B)** Energy dispersive X-ray emission spectra; Co:Zn ratio 1.94±0.04. **(C)** Transmission and reflection spectra and **(D)**atomic force microscopy image over sample etch (d= 35.7 nm).

IV. Raman Spectra and Analysis

The CCD x-axis was corrected for Raman shift using cyclohexane as a standard and correcting the associated pixels with a second order polynomial regression fitting. Raman spectra were corrected for grating and CCD efficiency, scattering cross section, and sample absorption. Published grating and CCD efficiencies for the Princeton Monochromators were used for instrumental corrections. To correct for scattering cross section of the samples, cyclohexane was collected after each sample scan. The most prominent peak at a Raman shift of 100 meV was integrated for each laser wavelength for the same accumulation time and compared to the theoretical value of the intensity of scattered light expected for each laser wavelength (Eq S1). From this ratio, we obtain a correction factor for each excitation wavelength that accounts for variations in laser power, solid angle collection, etc. Applying these correction factors to the regular Raman spectrum of cyclohexane recovers the expected λ^{-4} dependence of the scattering cross section (Figures S5A and S6B), confirming the efficacy of this correction procedure. These correction factors are then applied to the resonance Raman spectra collected for Co_3O_4 and $ZnCo_2O_4$ to obtain the data shown in Figures S5C and S6C. The scatter-corrected Raman spectra are then further corrected for differences in sample absorption at the various excitation wavelengths by dividing by the thickness-corrected absorption coefficient at the corresponding excitation wavelength. We correct the absorption coefficient for film thickness to account for the fact that the thickness of the films is greater than the penetration depth of most of the excitation wavelengths.

$$\text{Scat. Cross Section} = \text{Integrated area} \,/(h\nu)^4 \qquad \text{(S1)}$$

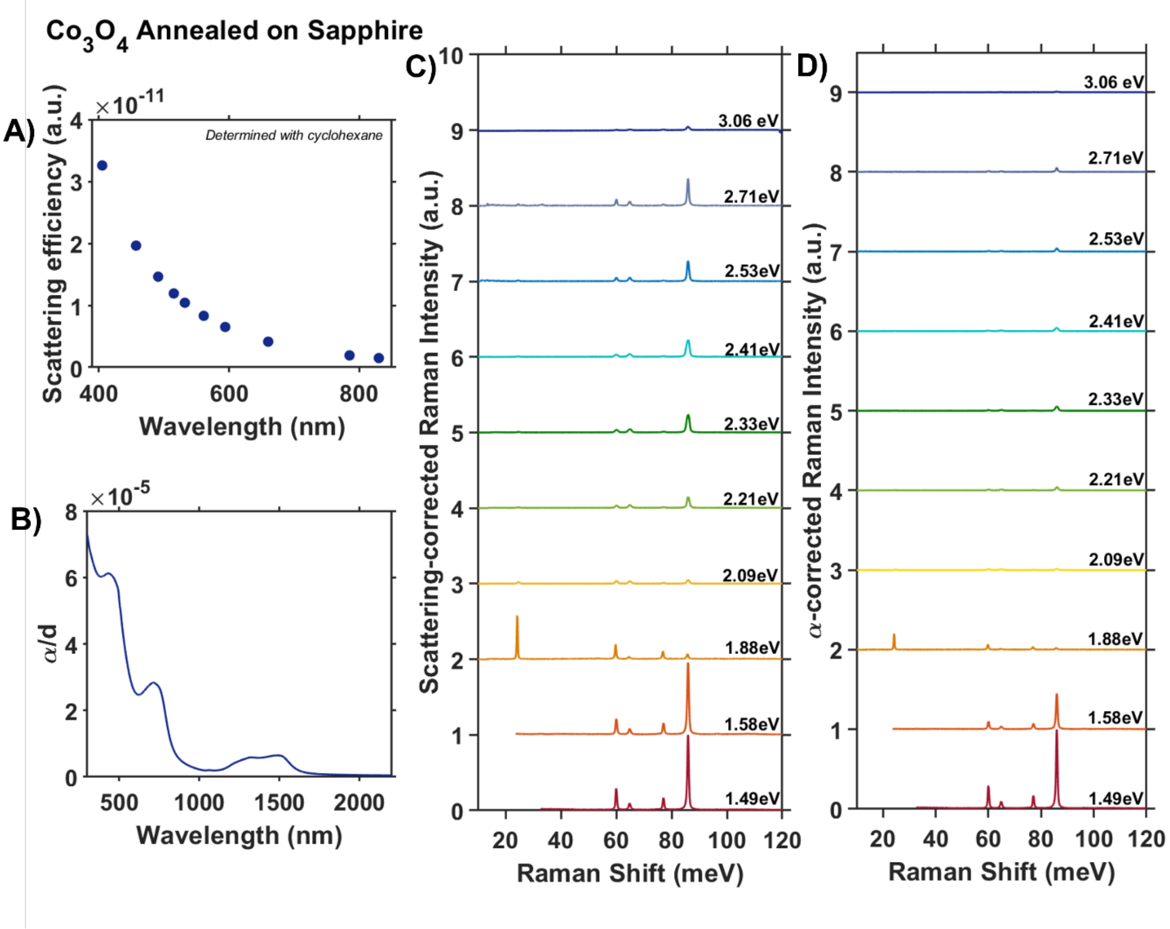

Figure S5. **(A)** Scattering cross section determined by cyclohexane spectra collected under the same parameters as sample spectra (Co_3O_4), **(B)** Absorption coefficient spectrum of Co_3O_4 corrected for thickness, **(C)** Raman profile of Co_3O_4 corrected for scattering cross section (offset for clarity) and **(D)** Raman profile of Co_3O_4 corrected for scattering cross section and sample absorption at each wavelength (offset for clarity). The data plotted in Figure 2C of the main text is sourced from **(D)**.

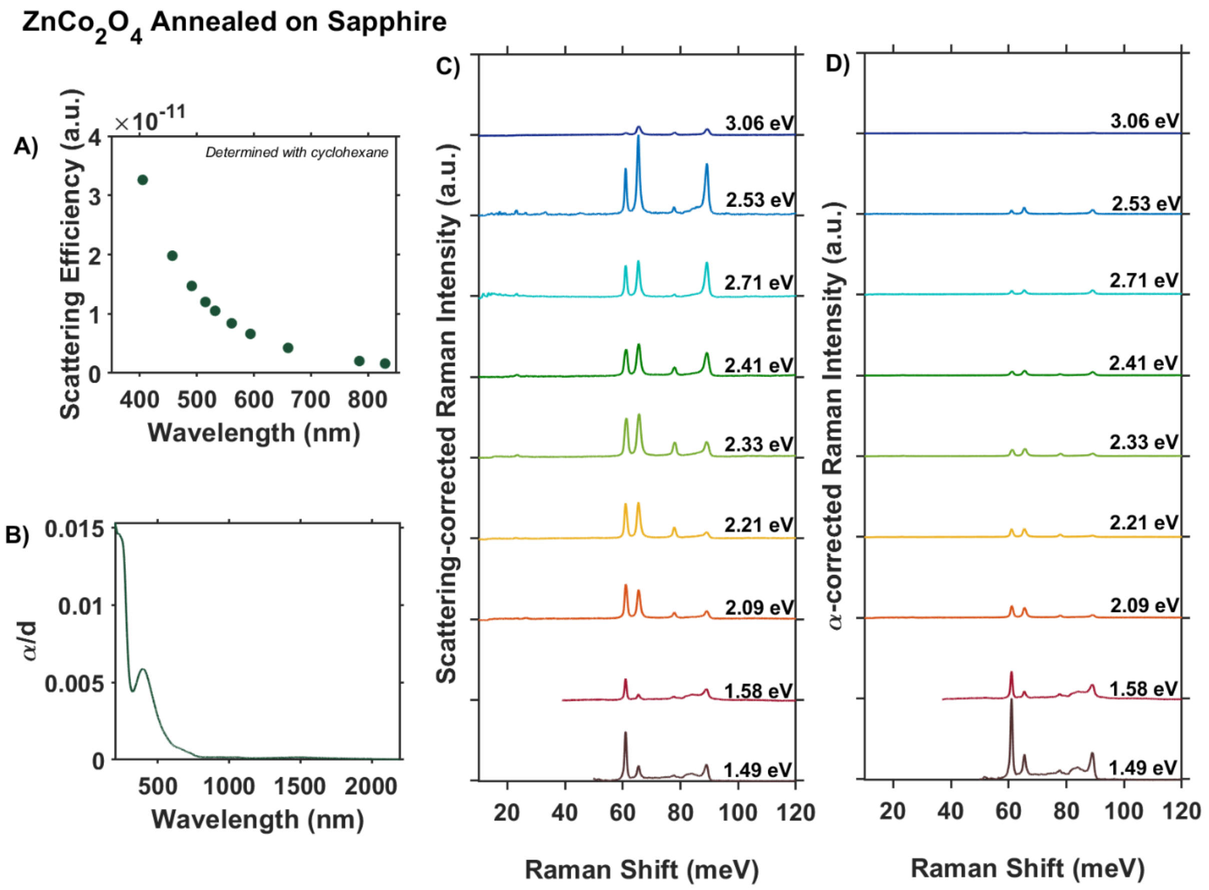

Figure S6. (A) Scattering cross section determined by cyclohexane spectra collected under the same parameters as sample spectra ($ZnCo_2O_4$), **(B)** absorption coefficient spectrum of $ZnCo_2O_4$ corrected for thickness, **(C)** Raman profile of $ZnCo_2O_4$ corrected for scattering cross section (offset for clarity) and **(D)** Raman profile of $ZnCo_2O_4$corrected for scattering cross section and sample absorption at each wavelength (offset for clarity). The data plotted in Figure 6C of the main text is sourced from **(D)**.

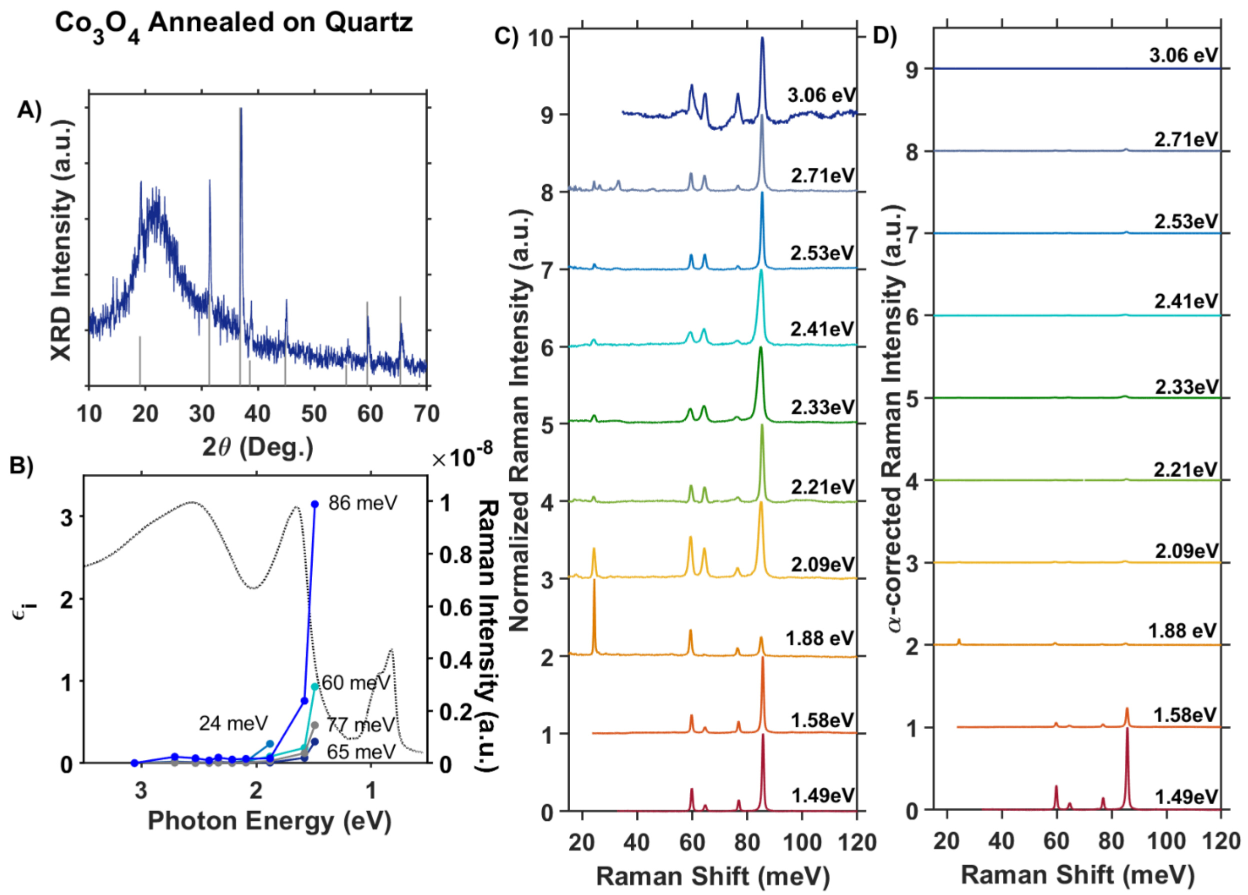

Figure S7. (A) X-ray diffraction pattern overlaid with a reference pattern for Co_3O_4 (ICDD 04-016-4508). **(B)** Raman intensity of Co_3O_4 phonon modes corrected for scattering cross section and sample absorption plotted versus excitation photon energy and overlaid with imaginary dielectric spectrum. **(C)** Internally normalized Co_3O_4 Raman spectra. **(D)** Co_3O_4 Raman spectra corrected for scattering cross section and sample absorption.

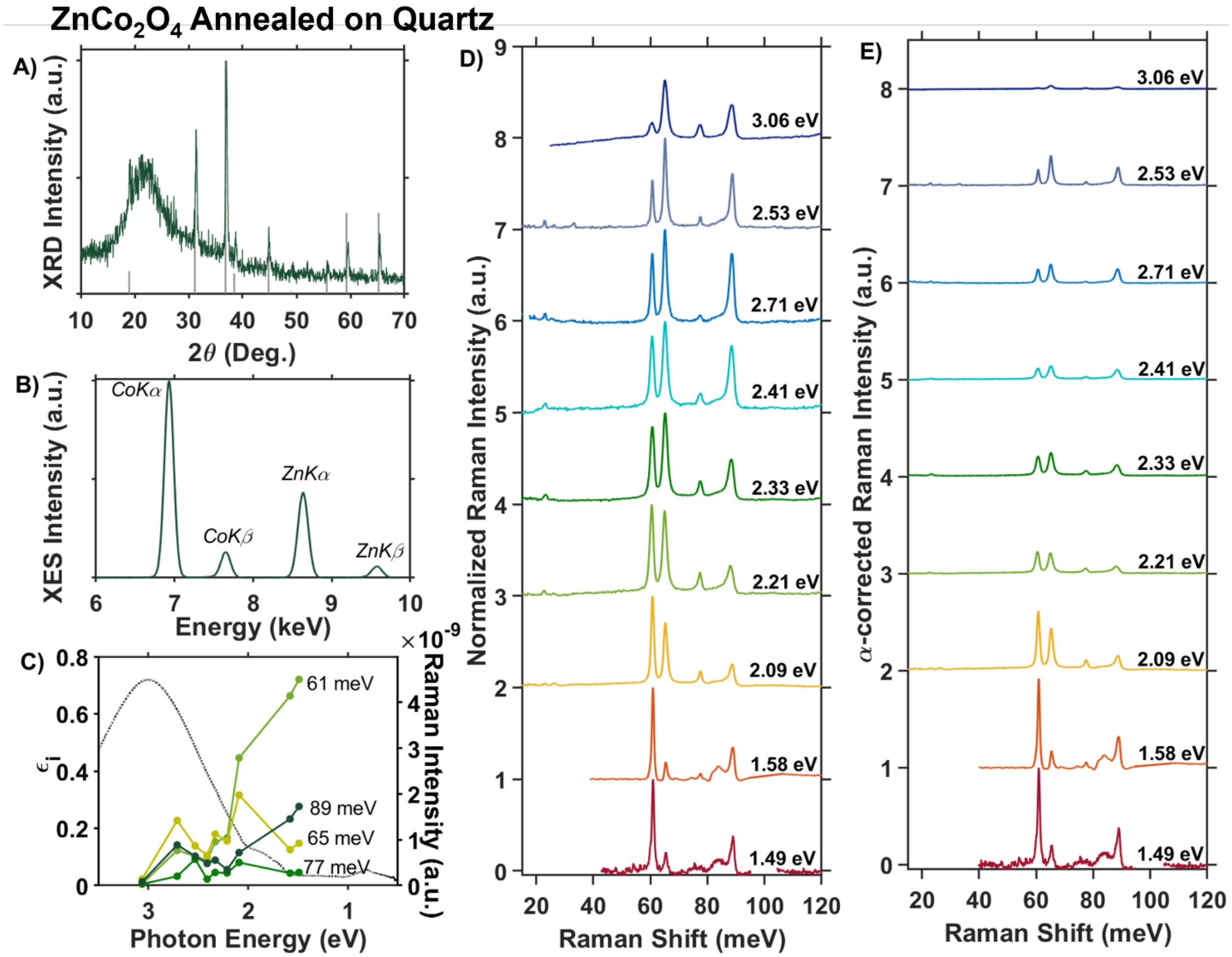

Figure S8. (A) X-ray diffraction pattern overlaid with a reference pattern for $ZnCo_2O_4$ (ICDD 00-023-1390), **(B)** Energy dispersive X-ray emission spectra; Co:Zn ratio 2.09 ± 0.001. **(C)** Raman intensity of $ZnCo_2O_4$ phonon modes corrected for scattering cross section and sample absorption plotted versus excitation photon energy and overlaid with imaginary dielectric spectra. **(D)** Internally normalized $ZnCo_2O_4$ Raman spectra. **(E)** $ZnCo_2O_4$ Raman spectra corrected for scattering cross section and sample absorption.

V. DFT correction parameters and primitive cells

The Hubbard and Hund parameters used in calculations are reported in Table S1. Figure S9 shows the primitive cells of the normal spinel oxides and the super cell of inverted $ZnCo_2O_4$. The crystallography data was sourced from Springer Materials.[17,18] The lattice parameter used for Co_3O_4 is 8.03999 Å, normal $ZnCo_2O_4$ is 8.00379 Å and after variable-cell relaxation there was no change to i$ZnCo_2O_4$ (x = 0.125); therefore, the same lattice parameter was used for iZnCo2O4 (a =8.00379 Å).

Table S1: Hubbard and Hund Parameters Applied to Co Spinel Materials for DFT Calculations.

Material	Hubbard U parameter	Hund J parameter
Co_3O_4	Co^{2+} (T_d): 3.36491492887531	Co^{2+} (T_d): 1.27814715775795
	Co^{3+} (O_h): 5.38486964497741	
$ZnCo_2O_4$	Co^{3+} (O_h): 5.35882728384708	
$ZnCo_2O_4$ (x=0.125)	Co^{3+} (O_h): 5.35882728384708	Co^{3+} (T_d): 1.27814715775795
	Co^{3+} (T_d): 3.36491492887531	

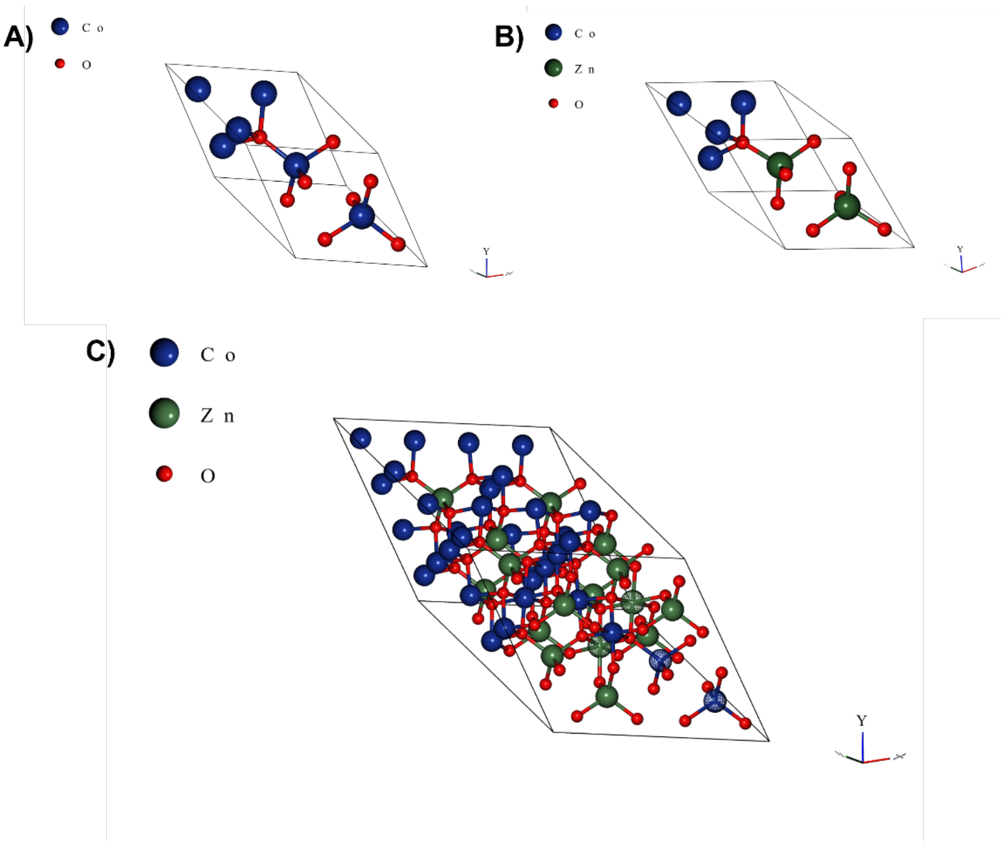

Figure S9. **(A)** Primitive cell of Co_3O_4. **(B)** Primitive cell of $ZnCo_2O_4$. **(C)** 2x2x2 super cell of i-$ZnCo_2O_4$ with two Co atoms in tetrahedral coordination and two Zn atoms in octahedral coordination.

VI. Computed Electronic Density of States

In Co_3O_4, the T_d Co atoms are antiferromagnetically coupled, as demonstrated by the opposite spin symmetry of the *e* and t_2 orbitals of the two atoms in the primitive cell (Figure S10A, B). Figures S10C and D plot the projected densities of states originating from the two different T_d Co^{2+} atoms (Figure S10A, B) and their nearest neighbors (4 oxygen atoms and 2 O_h Co^{3+} each). By plotting these atomic densities of states separately, spatial overlap in addition to energetic overlap among various orbital contributions becomes apparent. For example, the minimal O 2*p* character at the onset of the conduction band with a spin symmetry matching that of its neighboring T_d Co^{2+} *e* indicates that a metal-to-ligand-charge-transfer-type transition from the T_d Co^{2+} *e* state in the range of 0.8 – 1.6 eV is unlikely. In other words, when T_d Co^{2+} *e* is in the spin-up channel, the majority of O 2*p* character at the conduction band edge is in the spin down channel (as in Figure S10D). The converse is true for T_d Co^{2+} *e* in the spin-down channel. For this reason, metal-to-ligand charge transfer transitions are unlikely. Rather, we interpret the energetic overlap of O 2*p* and Co bands, which is most apparent in Figure 2D of the main text, to indicate covalency, where the metal-oxygen bonds act as a bridge allowing metal-to-metal charge transfer transitions to occur in the 0.8 – 1.6 eV energy range. By combining the energetic overlap information apparent in Figures 2D with the spatial overlap information apparent in Figure S10, we were able to assign the optical transitions present in the dielectric spectrum of Co_3O_4 (Figure 2C). These assignments are summarized in Table 1 of the main text.

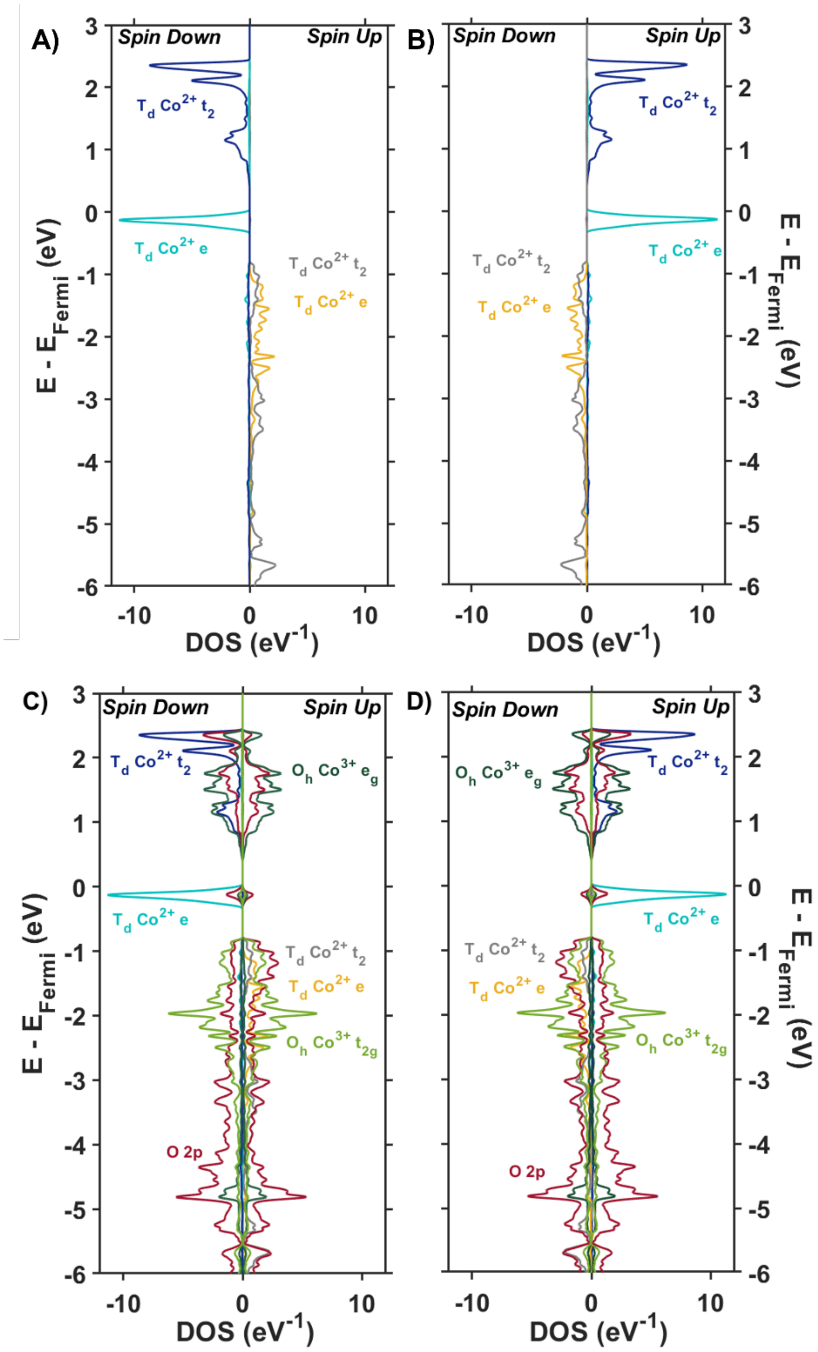

Figure S10. **(A)** The projected density of states of the 3*d* orbitals in one T_d Co^{2+} atom within the primitive cell of Co_3O_4. **(B)** The projected density of states of the 3*d* orbitals in the other T_d Co^{2+} atom in the primitive cell of Co_3O_4. **(C)** Projected density of states of one T_d Co atom and its neighboring 4 oxygen atoms and 2 O_h Co atoms. **(D)** Projected density of states of the other T_d Co atom and its neighboring 4 oxygen atoms and 2 O_h Co atoms.

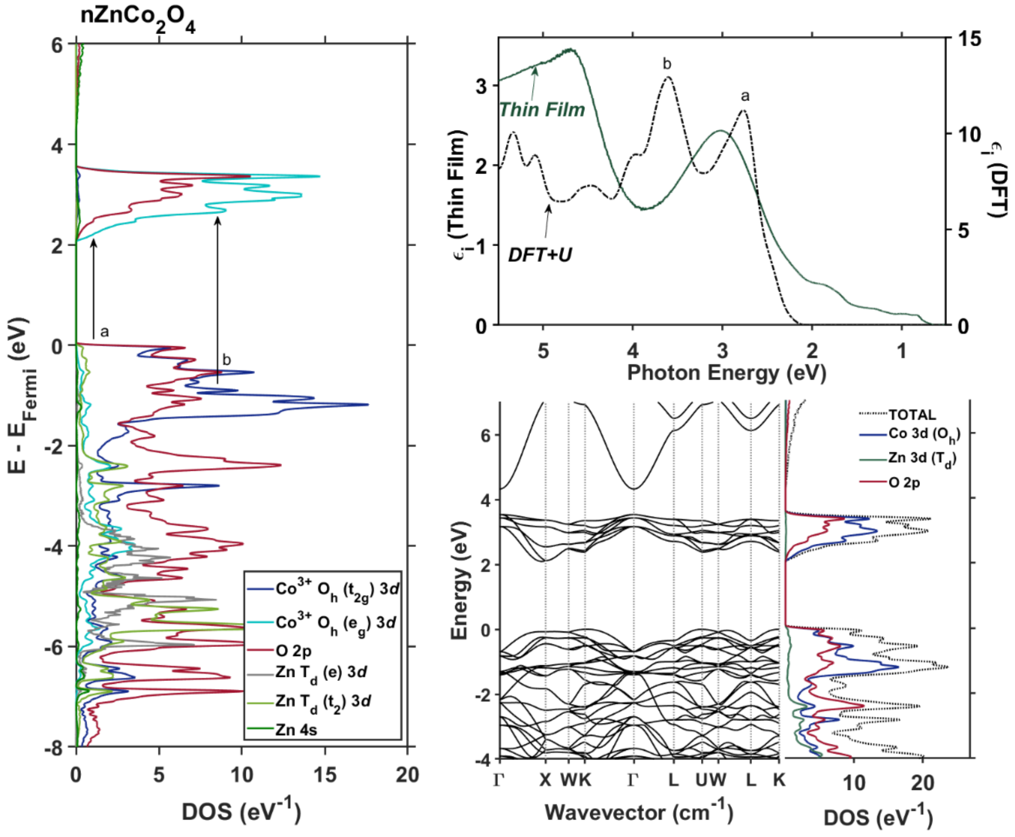

Figure S11. **(A)** Projected density of states of normal $ZnCo_2O_4$ with associated electronic assignments (a, b) labeled on **(B),** the dielectric spectrum of normal $ZnCo_2O_4$ calculated with DFT+*U* overlaid with the experimental dielectric spectrum of $ZnCo_2O_4$. **(C)** The band structure and projected density of states of normal $ZnCo_2O_4$.

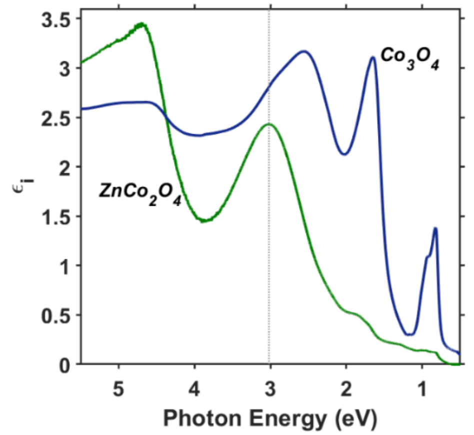

Figure S12. Imaginary dielectric spectrum of Co_3O_4 (blue) and $ZnCo_2O_4$ (green) with vertical line centered at the most prominent transition in $ZnCo_2O_4$, which matches the energy of the high-energy shoulder in Co_3O_4.

VII. $ZnCo_2O_4$ Annealing Study

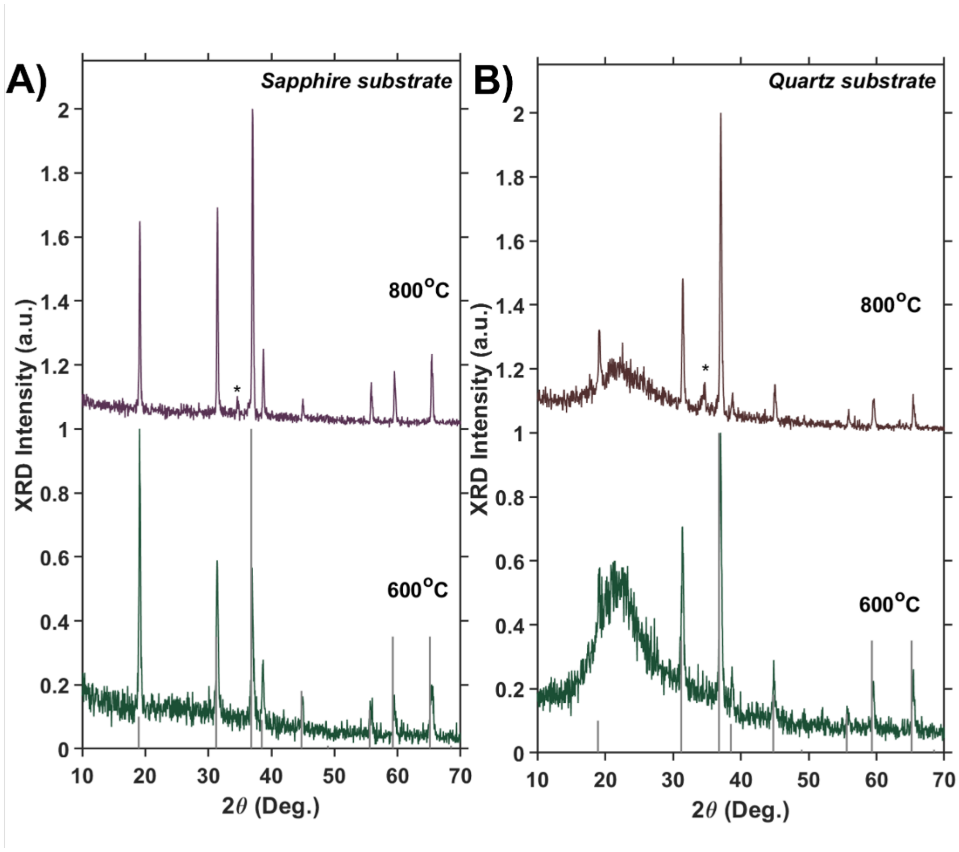

Figure S13. X-ray diffraction patterns of $ZnCo_2O_4$ thin films annealed on **(A)** sapphire at 800 °C (maroon) and 600 °C (green) and **(B)** quartz at the same temperatures. For both samples annealed at 800 °C a distinct peak at $2\theta = 34°$ appears, associated with wurtzite ZnO (ICDD 04-015-4060, asterisk) and not $ZnCo_2O_4$ (ICDD 00-023-1390; gray lines).

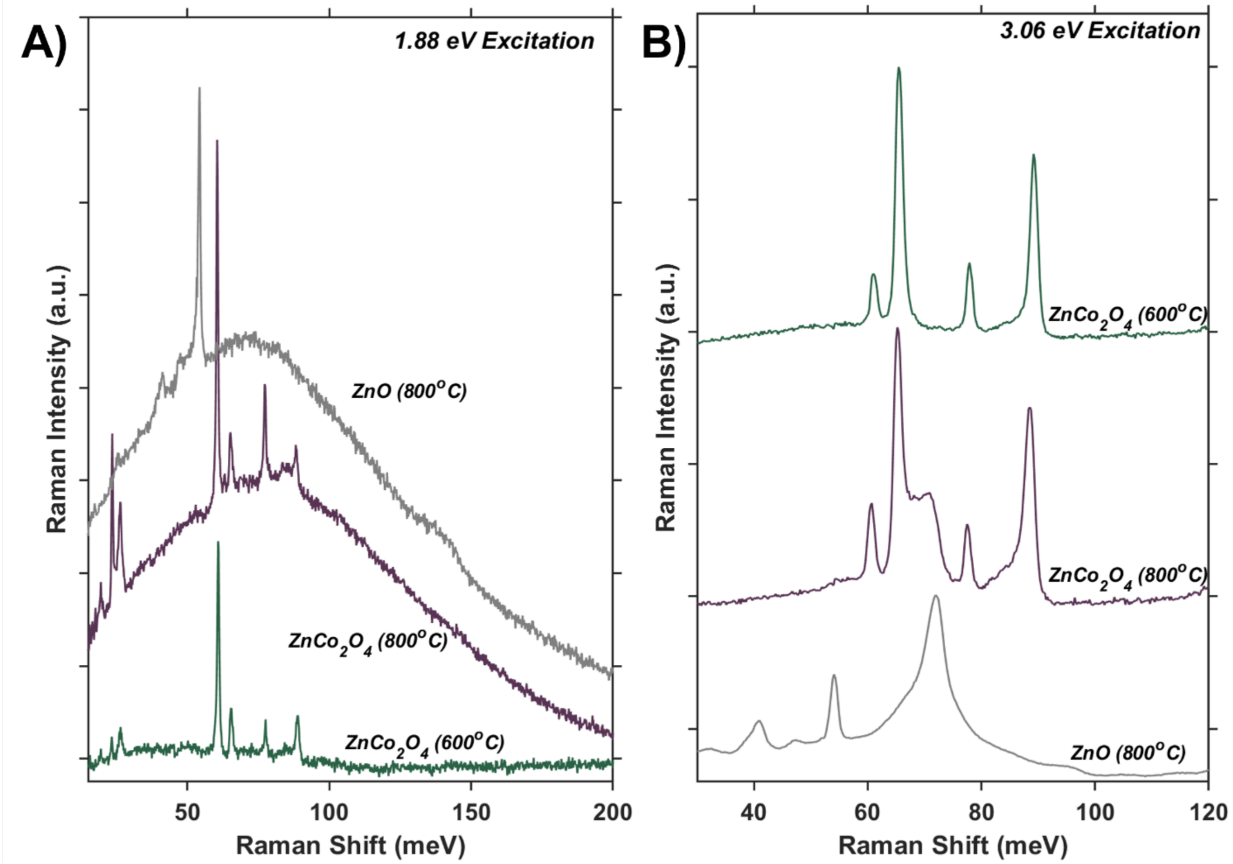

Figure S14. (A) Raman spectra of $ZnCo_2O_4$ annealed at 600 °C (green) and 800 °C (maroon) overlaid with a Raman spectrum of wurtzite ZnO collected with excitation energy of 1.88 eV (660 nm). The broad photoluminescent feature of ZnO is present in $ZnCo_2O_4$ when annealed at 800 °C but is not present in samples annealed at 600 °C. **(B)** Raman spectra of $ZnCo_2O_4$ annealed at 600 °C (green) and 800 °C (maroon) and wurtzite ZnO collected with excitation energy of 3.06 eV (405 nm). $ZnCo_2O_4$ annealed at 800 °C has a distinct shoulder at a Raman shift of ~70 meV, which matches the energy of the most intense feature in ZnO. The 70 meV shoulder is not present in $ZnCo_2O_4$ when annealed at 600 °C.

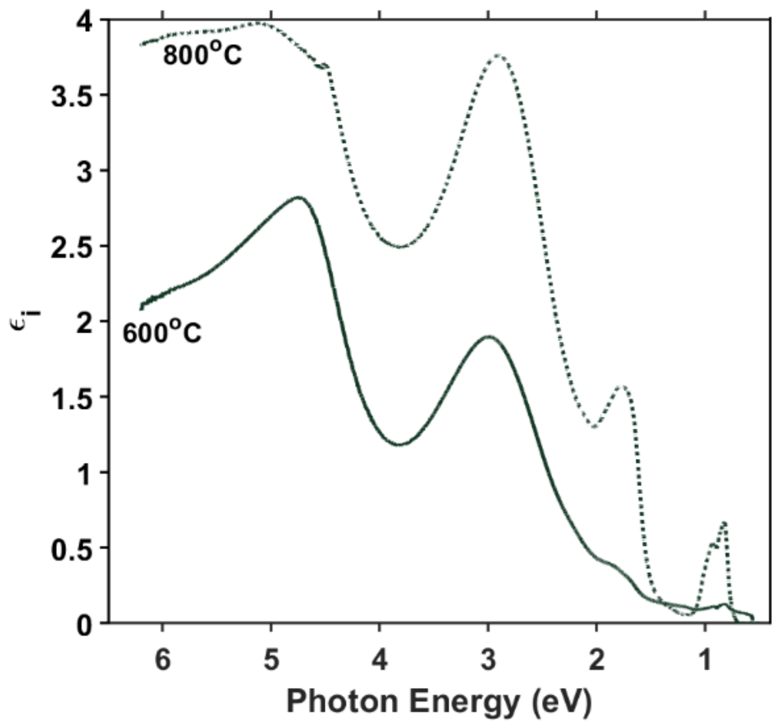

Figure S15. Imaginary dielectric spectra of $ZnCo_2O_4$ films annealed at 600°C (solid green) and 800 °C (dashed green). The features at ~0.8 and 1.6 eV are suppressed when $ZnCo_2O_4$ is annealed at 600°C.

VII. Temperature-dependent optical spectra of $ZnCo_2O_4$

The same approach to assessing temperature-dependence of optical transitions applied to Co_3O_4 was applied to $ZnCo_2O_4$. Spectra were collected above and below room temperature (294 K) and the difference in the imaginary dielectric spectrum was determined (Figure S16). While there are minor changes in intensity apparent in Figure S16 A,B, these changes often do not correlate with the energetic locations of features in the dielectric spectrum shown in Figure 6B. Furthermore, the intensities of the thermal difference spectra collected for Co_3O_4, (Figure 4A, B) are an order of magnitude larger than those observed in $ZnCo_2O_4$. This difference in intensity is further reflected in the integrated $\Delta\varepsilon_i$: the values observed for $ZnCo_2O_4$ are 10 times smaller than those determined for Co_3O_4. Additionally, the integrated TDS intensities of $ZnCo_2O_4$ are not strongly dependent on temperature. These observations lead to our conclusion that the optical transition observed $ZnCo_2O_4$ centered at 1.6 eV is not temperature-dependent.

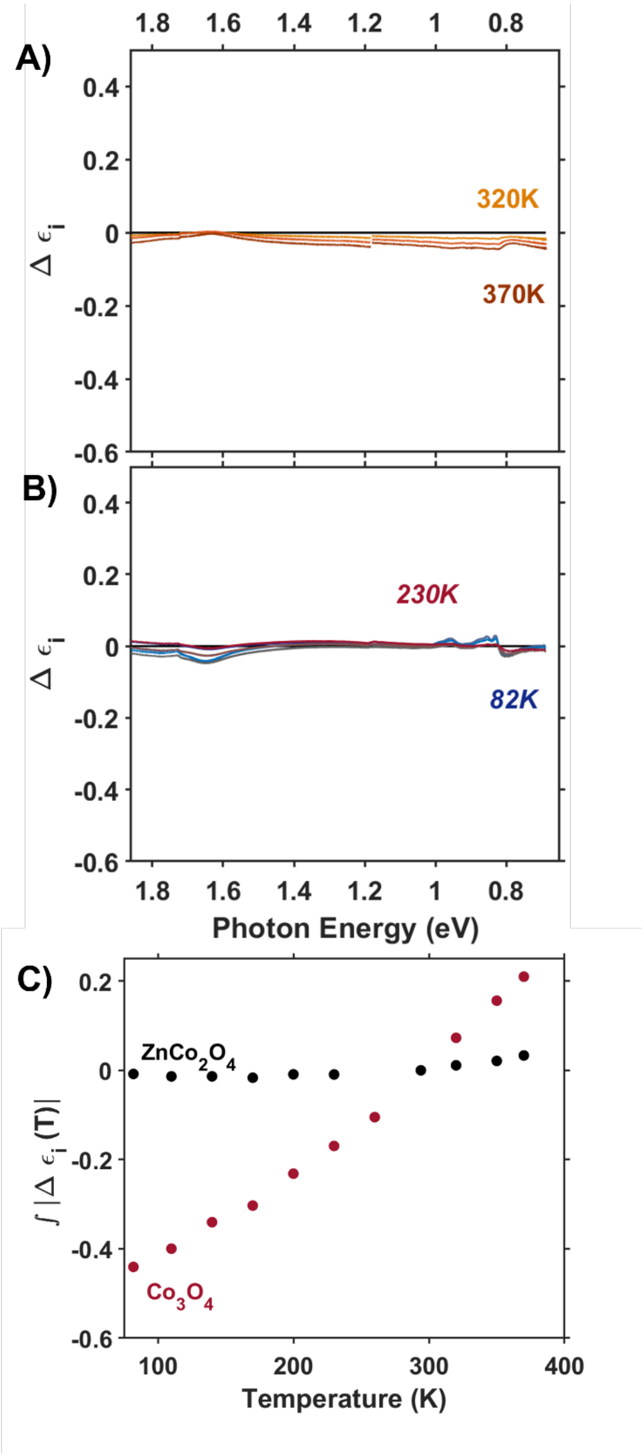

Figure S16. Thermal difference imaginary dielectric spectra of $ZnCo_2O_4$ collected at temperatures above (**A**) and below (**B**) room temperature (294 K). **C**) Intensity of the absolute value of the thermal difference spectra of $ZnCo_2O_4$ (black) and Co_3O_4 (red) integrated from 0.68 to 1.86 eV plotted against temperature in black (spectra collected below 294K are plotted as negative and above as positive).

Table S2: Phonon modes in Co_3O_4 at Γ

Phonon Energy (meV)	Vibrational Motion in Primitive Cell	Raman/IR Active
23.93019	Symmetric T_d Co motion	Raman
23.93019 (x2)		
27.67823 (x2)		IR
28.39114		IR
31.14235 (x3)		IR
44.57232 (x2)		
48.26705 (x2)		IR
48.48402		IR
58.00973 (x3)		
58.33085	O_h Co breathing	Raman
61.28787 (x2)		
64.95036	Asymmetric oxygen breathing about T_d Co	Raman
64.95036		
65.53681 (x3)		IR
66.78409 (x3)		
71.68518 (x2)		IR
76.34575	Asymmetric oxygen breathing about T_d Co	Raman
76.37427 (x2)		IR
76.96691		IR
82.25856 (x2)		IR
82.73218		
83.89267	Oxygen breathing about T_d Co	Raman
85.40527		IR

Table S3: Phonon modes in Normal $ZnCo_2O_4$ at Γ

Phonon Energy (meV)	Vibrational Motion in Primitive Cell	Raman/IR Active
20.0842 (x2)		IR
21.43191		IR
22.39527 (x3)		
23.24332	Symmetric Zn motion	Raman
23.24332 (x2)		
47.58018 (x2)		
47.66077 (x2)		IR
48.10091		IR
59.18386 (x3)		
60.16829	O_h Co breathing	Raman
62.01442	Asymmetric oxygen breathing about T_d Co	Raman
62.01442		
66.61671 (x2)		
66.8312 (x3)		
67.48088		
73.99129 (x2)		IR
77.29299	Asymmetric oxygen breathing about T_d Co	Raman
77.29299 (x2)		
78.46588		IR
83.71289 (x2)		IR
86.66743		
88.41809	Symmetric oxygen breathing about Zn	Raman
88.43049		IR

References

(1) Shelton, J. L.; Knowles, K. E. Thermally Activated Optical Absorption into Polaronic States in Hematite. *J. Phys. Chem. Lett.* **2021**, *12*, 3343–3351.

(2) Barybin, A.; Shapovalov, V. Substrate Effect on the Optical Reflectance and Transmittance of Thin-Film Structures. *Int. J. Opt.* **2010**, *2010*, 1–18.

(3) Giannozzi, P.; Baroni, S.; Bonini, N.; Calandra, M.; Car, R.; Cavazzoni, C.; Ceresoli, D.; Chiarotti, G. L.; Cococcioni, M.; Dabo, I.; Dal Corso, A.; De Gironcoli, S.; Fabris, S.; Fratesi, G.; Gebauer, R.; Gerstmann, U.; Gougoussis, C.; Kokalj, A.; Lazzeri, M.; Martin-Samos, L.; Marzari, N.; Mauri, F.; Mazzarello, R.; Paolini, S.; Pasquarello, A.; Paulatto, L.; Sbraccia, C.; Scandolo, S.; Sclauzero, G.; Seitsonen, A. P.; Smogunov, A.; Umari, P.; Wentzcovitch, R. M. QUANTUM ESPRESSO: A Modular and Open-Source Software Project for Quantum Simulations of Materials. *J. Phys.: Condens. Matter* **2009**, *21*, 395502.

(4) Giannozzi, P.; Andreussi, O.; Brumme, T.; Bunau, O.; Buongiorno Nardelli, M.; Calandra, M.; Car, R.; Cavazzoni, C.; Ceresoli, D.; Cococcioni, M.; Colonna, N.; Carnimeo, I.; Dal Corso, A.; De Gironcoli, S.; Delugas, P.; DiStasio, R. A.; Ferretti, A.; Floris, A.; Fratesi, G.; Fugallo, G.; Gebauer, R.; Gerstmann, U.; Giustino, F.; Gorni, T.; Jia, J.; Kawamura, M.; Ko, H.-Y.; Kokalj, A.; Küçükbenli, E.; Lazzeri, M.; Marsili, M.; Marzari, N.; Mauri, F.; Nguyen, N. L.; Nguyen, H.-V.; Otero-de-la-Roza, A.; Paulatto, L.; Poncé, S.; Rocca, D.; Sabatini, R.; Santra, B.; Schlipf, M.; Seitsonen, A. P.; Smogunov, A.; Timrov, I.; Thonhauser, T.; Umari, P.; Vast, N.; Wu, X.; Baroni, S. Advanced Capabilities for Materials Modelling with Quantum ESPRESSO. *J. Phys.: Condens. Matter* **2017**, *29*, 465901.

(5) Giannozzi, P.; Baseggio, O.; Bonfà, P.; Brunato, D.; Car, R.; Carnimeo, I.; Cavazzoni, C.; De Gironcoli, S.; Delugas, P.; Ferrari Ruffino, F.; Ferretti, A.; Marzari, N.; Timrov, I.; Urru, A.; Baroni, S. Q UANTUM ESPRESSO toward the Exascale. *J. Chem. Phys.* **2020**, *152*, 154105.

(6) Perdew, J. P.; Burke, K.; Ernzerhof, M. Generalized Gradient Approximation Made Simple. *Phys. Rev. Lett.* **1996**, *77*, 3865–3868.

(7) Perdew, J. P.; Ruzsinszky, A.; Csonka, G. I.; Vydrov, O. A.; Scuseria, G. E.; Constantin, L. A.; Zhou, X.; Burke, K. Restoring the Density-Gradient Expansion for Exchange in Solids and Surfaces. *Phys. Rev. Lett.* **2008**, *100*, 136406.

(8) Hamann, D. R. Optimized Norm-Conserving Vanderbilt Pseudopotentials. *Phys. Rev. B* **2013**, *88*, 085117.

(9) Van Setten, M. J.; Giantomassi, M.; Bousquet, E.; Verstraete, M. J.; Hamann, D. R.; Gonze, X.; Rignanese, G.-M. The PseudoDojo: Training and Grading a 85 Element Optimized Norm-Conserving Pseudopotential Table. *Comput. Phys. Commun.* **2018**, *226*, 39–54.

(10) *Practical Methods of Optimization*, 2nd ed.; Fletcher, R., Ed.; Wiley: Chichester New York, 2010.

(11) Billeter, S. R.; Turner, A. J.; Thiel, W. Linear Scaling Geometry Optimisation and Transition State Search in Hybrid Delocalised Internal Coordinates. *Phys. Chem. Chem. Phys.* **2000**, *2*, 2177–2186.

(12) Billeter, S. R.; Curioni, A.; Andreoni, W. Efficient Linear Scaling Geometry Optimization and Transition-State Search for Direct Wavefunction Optimization Schemes in Density Functional Theory Using a Plane-Wave Basis. *Computational Materials Science* **2003**, *27*, 437–445.

(13) Cococcioni, M.; De Gironcoli, S. Linear Response Approach to the Calculation of the Effective Interaction Parameters in the LDA + U Method. *Phys. Rev. B* **2005**, *71*, 035105.

(14) Himmetoglu, B.; Wentzcovitch, R. M.; Cococcioni, M. First-Principles Study of Electronic and Structural Properties of CuO. *Phys. Rev. B* **2011**, *84*, 115108.

(15) Shelton, J. L.; Knowles, K. E. Polaronic Optical Transitions in Hematite (α-Fe_2O_3) Revealed by First-Principles Electron–Phonon Coupling. *J. Chem. Phys.* **2022**, *157*, 174703.

(16) Roth, W. L. The Magnetic Structure of Co_3O_4. *J. Phys. Chem. Solids* **1964**, *25*, 1–10.

(17) Smith, W.; Hobson, A. The Structure of Cobalt Oxide, Co_3O_4. *Acta Crystallographica, Section B: Structural Crystallography and Crystal Chemistry* **1973**, *29*, 362–363.

(18) Krezhov, K.; Konstantinov, P. On the Cationic Distribution in Zinc-Cobalt Oxide Spinels. *J. Phys.: Condens. Matter* **1993**, 9287.
